# AsrR Is an Oxidative Stress Sensing Regulator Modulating *Enterococcus faecium* Opportunistic Traits, Antimicrobial Resistance, and Pathogenicity

**DOI:** 10.1371/journal.ppat.1002834

**Published:** 2012-08-02

**Authors:** François Lebreton, Willem van Schaik, Maurizio Sanguinetti, Brunella Posteraro, Riccardo Torelli, Florian Le Bras, Nicolas Verneuil, Xinglin Zhang, Jean-Christophe Giard, Anne Dhalluin, Rob J. L. Willems, Roland Leclercq, Vincent Cattoir

**Affiliations:** 1 University of Caen Basse-Normandie, EA4655 (team “Antibioresistance”), Medical School, Caen, France; 2 University Medical Center Utrecht, Department of Medical Microbiology, Utrecht, The Netherlands; 3 Catholic University of Sacred Heart, Institute of Microbiology, Rome, Italy; 4 Catholic University of Sacred Heart, Institute of Hygiene, Rome, Italy; 5 University of Caen Basse-Normandie, EA4655 (team “Stress and Virulence”), Caen, France; 6 University Hospital of Caen, Department of Microbiology, Caen, France; University of California, San Francisco, United States of America

## Abstract

Oxidative stress serves as an important host/environmental signal that triggers a wide range of responses in microorganisms. Here, we identified an oxidative stress sensor and response regulator in the important multidrug-resistant nosocomial pathogen *Enterococcus faecium* belonging to the MarR family and called AsrR (antibiotic and stress response regulator). The AsrR regulator used cysteine oxidation to sense the hydrogen peroxide which results in its dissociation to promoter DNA. Transcriptome analysis showed that the AsrR regulon was composed of 181 genes, including representing functionally diverse groups involved in pathogenesis, antibiotic and antimicrobial peptide resistance, oxidative stress, and adaptive responses. Consistent with the upregulated expression of the *pbp5* gene, encoding a low-affinity penicillin-binding protein, the *asrR* null mutant was found to be more resistant to β-lactam antibiotics. Deletion of *asrR* markedly decreased the bactericidal activity of ampicillin and vancomycin, which are both commonly used to treat infections due to enterococci, and also led to over-expression of two major adhesins, *acm* and *ecbA*, which resulted in enhanced *in vitro* adhesion to human intestinal cells. Additional pathogenic traits were also reinforced in the *asrR* null mutant including greater capacity than the parental strain to form biofilm *in vitro* and greater persistance in *Galleria mellonella* colonization and mouse systemic infection models. Despite overexpression of oxidative stress-response genes, deletion of *asrR* was associated with a decreased oxidative stress resistance *in vitro*, which correlated with a reduced resistance to phagocytic killing by murine macrophages. Interestingly, both strains showed similar amounts of intracellular reactive oxygen species. Finally, we observed a mutator phenotype and enhanced DNA transfer frequencies in the *asrR* deleted strain. These data indicate that AsrR plays a major role in antimicrobial resistance and adaptation for survival within the host, thereby contributes importantly to the opportunistic traits of *E. faecium*.

## Author Summary

Multiple antibiotic-resistant isolates of the opportunistic pathogen *Enterococcus faecium* have emerged and spread worldwide. However, studies aimed at identifying mechanisms that underlie the transformation of *E. faecium* from its commensal nature into a nosocomial pathogen are scarce. We report pleiotropic roles for a novel oxidative-sensing regulator, called AsrR (antibiotic and stress response regulator), in *E. faecium*. Based on transcriptomic analysis, phenotypic studies, and animal models, we demonstrate that *asrR* deletion is responsible for i) diminished susceptibility to penicillins, vancomycin, and cationic antimicrobial peptides, ii) increased adhesion to human cells and biofilm formation, iii) a mutator phenotype and enhanced DNA transfer frequencies, iv) decreased resistance to oxidative stress both *in vitro* and in murine macrophages, and v) increased host-persistence in both insect and mouse models. AsrR is a stress-sensor and is promptly inactivated in the presence of hydrogen peroxide. Therefore, oxidative stress, which is a main challenge during infection, may be a significant signal used by *E. faecium* to promote opportunistic traits. This provides a significant resource combining, for the first time in *E. faecium*, a global transcriptomic approach and a thorough phenotypic study, which places AsrR as a key regulator modulating pathogenicity, antimicrobial resistance, and environmental adaptation.

## Introduction

Enterococci are commensal Gram-positive cocci of intestinal origin. First reported as a cause of infective endocarditis in 1899, enterococci have also become, over the past 20 years, the 2^nd^–3^rd^ most common organisms isolated from healthcare-associated infections [1], [2]. In the USA, the emergence of enterococci as nosocomial pathogens was associated with a gradual replacement of *Enterococcus faecalis* by *Enterococcus faecium* and an epidemic spread of vancomycin-resistant *E. faecium* [3]. Acquisition of resistance to ampicillin and then to vancomycin, impacting the antibiotic treatments of choice, has been assumed to be the major factor responsible for transforming this organism from its docile, commensal nature into a significant nosocomial pathogen [3]. Reports on the transfer of vancomycin resistance from enterococci to methicillin-resistant *Staphylococcus aureus* stress the need to better understand the molecular epidemiology, as well as the transmissibility and virulence of enterococci, to control further spread and develop treatment and eradication strategies [4], [5].

Mortality associated with vancomycin-resistant *E. faecium* infections is high but is more related to severe underlying diseases in infected patients than to production of bacterial virulence factors [6]. One of the most remarkable features of *E. faecium* isolates is their striking capacity to colonize both healthy carriers and patients, to survive to the host defences and to spread in the hospital environment, leading to major outbreaks [7]. The factors underlying its colonization capacities, including host-persistence, environmental stress response and adaptation, are only poorly understood.

In addition to antibiotic resistance genes, several virulence genes have been identified in *E. faecium* of which *esp_Efm_* and *acm* (encoding a surface protein and a collagen adhesin, respectively) have been experimentally proven to be important for infection in animal models [8], [9]. In numerous organisms, virulence genes are controlled by environmental stresses and involve alternative σ factors of RNA polymerase and specific transcriptional regulators. Enterococci lack a σ^B^-like general stress σ factor, but approximately 10 transcriptionnal regulators have been shown to be involved in virulence and stress response in the related bacterium *E. faecalis* [10]–[13]. Deciphering the regulatory pathways that lead to virulence and antibiotic resistance is crucial to understand the mechanisms by which *E. faecium* can colonize and infect critically ill patients.

MarR family transcriptional regulators play key roles in several bacterial species, including SarA, MgrA, and their homologs in *Staphylococcus aureus* [14]–[18]. These regulators utilize cysteine oxidation to sense oxidative stress and regulate bacterial responses. The MarR sub-family of OhrR (organic hydroperoxide resistance regulator) regulators, found in *Bacillus subtilis* and in numerous other Gram-positives, regulate bacterial resistance to organic hydroperoxides using similar redox-sensing mechanisms [19]–[23]. Interestingly, in pathogenic bacteria such as *S. aureus* and *Pseudomonas aeruginosa*, MarR regulators seem to play broad regulatory roles that have profound effects on global properties of the pathogen. MgrA (multiple gene regulator A) is the first example of utilizing this mechanism to regulate antibiotic resistance and expression of virulence factors in *S. aureus* [24], [25]. In recent work, the MarR family transcriptional regulator OspR (oxidative stress response and pigment production regulator), homologous to MgrA, was found to play key roles in antibiotic resistance and virulence regulation in *P. aeruginosa* [26]. These discoveries raise the possibility that the opportunistic microorganism *E. faecium* may also harbor a MgrA/OspR homologue that could assume global roles in pathogenesis through sensing oxidative stress.

We report the finding of a MarR family oxidative sensing regulator, AsrR (antibiotic and stress response regulator), in *E. faecium*. A search for MgrA/OspR homologues in *E. faecium* identified AsrR that shares significant sequence identities with OspR and OhrR proteins. AsrR was found to possess the winged-helix DNA binding motif and the two cysteine residues present in the MarR family members and to exert a global regulatory role on adaptive responses, antimicrobial resistance, oxidative stress response, autolysis, and pathogenicity in *E. faecium*. These results should help shed light on the understanding of the multifaceted adaptative response in *E. faecium* and its remarkable colonizing capacities.

## Results

### *S. aureus* MgrA/*P. aeruginosa* OspR homolog in *E. faecium*

**Figure 1:**
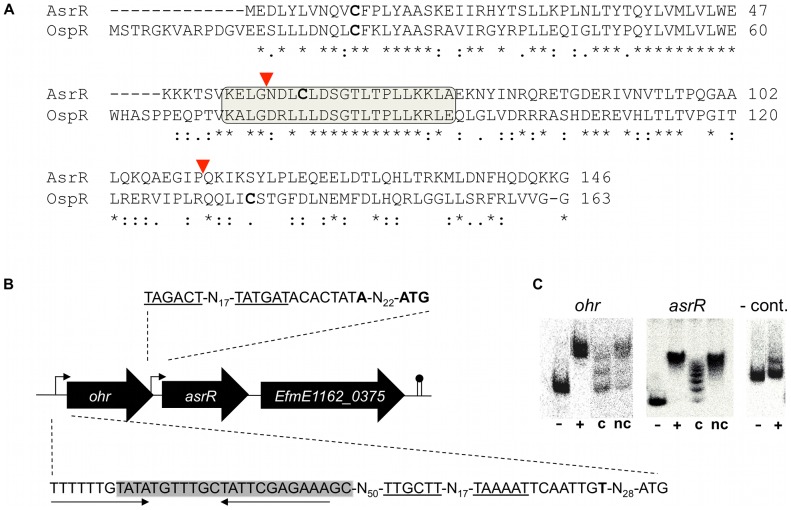
Deduced structure, genomic environment, and DNA binding of AsrR. (A) Comparison of *E. faecium* AsrR and *P. aeruginosa* OspR (Accession number NP_251515.1) amino acid sequences. Asterisks, colons and periods indicate identical, strongly similar, and weakly similar residues, respectively. The winged helix-turn-helix motif is boxed. Cysteine residues, found in AsrR and OspR sequences, are indicated in bold-face characters. The region comprised between red triangles corresponds to the deletion of AsrR protein (*ca.* 42%) in the Δ*asrR* strain. (B) Genetic context of the *asrR* locus. Broken arrows indicate the mapped promoters, start codons and transcription start sites in HM1070 are indicated in bold-face characters, conserved −10 and −35 motifs are underlined. The AsrR binding box, identified experimentally by footprinting (Figure S1C), is boxed on the *ohr* promoter sequence and putative inverted repeats are highlighted by arrows. The ORF number is indicated according to the strain E1162 annotation (Genbank accession ABQJ00000000). (C) Gel shift experiments showing binding of His_6_-AsrR to the *ohr* and *asrR* promoters. Binding of AsrR was evaluated without (−) or with (+) purified His_6_-tagged AsrR protein. Specificity of the interaction was evaluated using a control DNA (-cont.) amplified from a non-promoter region and in the presence of unlabelled competitor (c) or non-competitor DNA (nc).

The global regulators MgrA of *S. aureus* and OspR of *P. aeruginosa* play key roles in virulence regulation [15], [16], [26]. Using BLASTP analysis, we identified a MgrA/OspR homologue in the genome sequence of the *E. faecium* E1162 clinical isolate [7]. A single significant hit was obtained with the deduced protein of *EfmE1162_0374* showing 34% and 44% amino acid identity with MgrA and OspR, respectively. After further study, we renamed *EfmE1162_0374* as *asrR* (for antibiotic and stress response regulator) based on the observed phenotypes presented below. Pfam analysis showed that the deduced AsrR protein possessed the MarR-type helix–turn–helix motif placing it in the MarR protein family (Figure 1A). Similarly to OspR, AsrR harbors two cysteine residues, found at positions 11 and 61 (Figure 1A). These residues have been shown to play a major role in oxidative stress sensing in OspR [26]. Sequence comparison showed that *asrR* was conserved among all *E. faecium* isolates and that *asrR* putative homologs were present in *Enterococcus gallinarum* and *Enterococcus casseliflavus* but not in *E. faecalis* (data not shown).

### Organization of the *asrR* transcriptional unit

Fifty-six base pairs upstream of *asrR*, the *EfmE1162_0373* locus, subsequently renamed *ohr*, encoded a putative protein highly similar to Ohr proteins described in numerous Gram-positive bacteria (Figure 1B). Usually, *ohr* is part of a two-gene operon and is co-transcribed with an upstream adjacent gene *ohrR* encoding a transcriptional regulator [20], [23], [27]. This organization was not found in *E. faecium* since no *ohrR* homologue was found upstream of the *EfmE1162_0373* locus. However, the homology of *asrR* with *ohrR* (42% nucleotide identity) suggested that AsrR may control the expression of *ohr*. RACE-PCR experiments in *E. faecium* HM1070 identified one promoter upstream of both *asrR* and *ohr* genes, and we showed that cotranscription of *ohr* and *asrR* may occur from the *ohr* promoter (Figure 1B, Figure S1A, Figure S1B). We also determined experimentally the AsrR binding site upstream of the *ohr* gene (Figure 1B, Figure S1C). A putative AT-rich inverted repeat sequence was found that overlapped the AsrR binding box of *ohr* (Figure 1B), which is consistent with the fact that proteins of the MarR family specifically bind palindromic or pseudopalindromic sites using a conserved winged helix fold [22], [28]. The direct interaction of AsrR with the *ohr* and *asrR* promoters was tested by electromobility shift assay (EMSA) (Figure 1C). Purified His_6_-tagged AsrR bound specifically to the *ohr* and *asrR* promoter sequences, while failing to shift a non-promoter DNA fragment used as a control (Figure 1C). In addition, the binding was lost in the presence of unlabelled competitor and restored in the presence of non-competitor DNA (Figure 1C). Finally, 69 bp downstream of *asrR*, *EfmE1162_0375* encoded a putative permease of unknown function conserved among enterococci (Figure 1B).

### Oxidative stress inactivates AsrR and modulates *asrR* and *ohr* expression

Suspecting that *asrR* expression was modulated by oxidative stress, we used quantitative real-time PCR (qRT-PCR) to analyze the expression of *asrR* in *E. faecium* HM1070 after 10 or 20 min of a 2 mM hydrogen peroxide (H_2_O_2_) challenge (Figure S2). In addition, we also analyzed the expression of *ohr* since AsrR interacts directly with the promoter of this gene (see above). We observed a strong induction of expression of both genes after 10 min of H_2_O_2_ treatment. Induction was higher for the *ohr* gene and decreased similarly for both genes after 20 min of H_2_O_2_ challenge (Figure S2). By contrast, no *ohr* upregulation was found in response to H_2_O_2_ oxidative stress in the Δ*asrR* mutant strain (data not shown).

**Figure 2:**
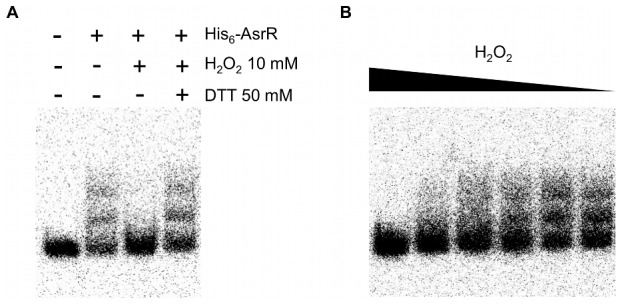
Inhibition of AsrR binding to DNA by H_2_O_2_. (A) Gel shift experiments showing binding of His_6_-tagged AsrR to the *ohr* promoter. Binding of AsrR was evaluated without (−) or with (+) purified His_6_-tagged AsrR protein, 10 mM of H_2_O_2_, and 50 mM of DTT. (B) Concentration-dependent binding of purified His_6_-tagged AsrR protein in the presence of different concentrations of H_2_O_2_ from 10 to 0.01 mM. The His_6_-tagged AsrR purified protein was found to be inactivated by H_2_O_2_ and then lost its capacity to bind the *ohr* promoter. This concentration-dependent inactivation of H_2_O_2_ was found to be reversible by the addition of DTT.

As suspected, we showed that AsrR was a functionnal sensor of the oxidative stress. Indeed, we showed by using EMSA that after a treatment with 10 mM of H_2_0_2_ the oxidized His_6_-tagged AsrR was no longer able to bind to the *ohr* promoter (Figure 2A). In addition, this effect was reversible since the addition of a reducing agent (i.e. DTT) restored the binding ability of the AsrR protein (Figure 2A), and it was also dependent of the H_2_O_2_ concentration (Figure 2B).

### Identification of AsrR-regulated genes

To identify the set of AsrR-regulated genes in *E. faecium*, the transcriptome of the Δ*asrR* mutant was compared to those of the *E. faecium* HM1070 parental strain and of the knock-in Δ*asrR*::*asrR* complemented strain. Since the *E. faecium* microarray was custom-made based on the E1162 genome, an *in silico* comparative genomic hybridization was performed between HM1070 (entirely sequenced, unpublished) and E1162 *E. faecium* genomes. We found that 73.5% of probes (3924 of a total of 5337) designed for E1162 had 100% identical targets in HM1070 DNA. In addition, 6.8% of probes (364 of a total of 5337) had only one or two mismatches. Therefore, the E1162 *E. faecium* microarray appeared to be suitable for HM1070 transcriptome analysis since around 80% of the probes were conserved in both genomes.

**Figure 3:**
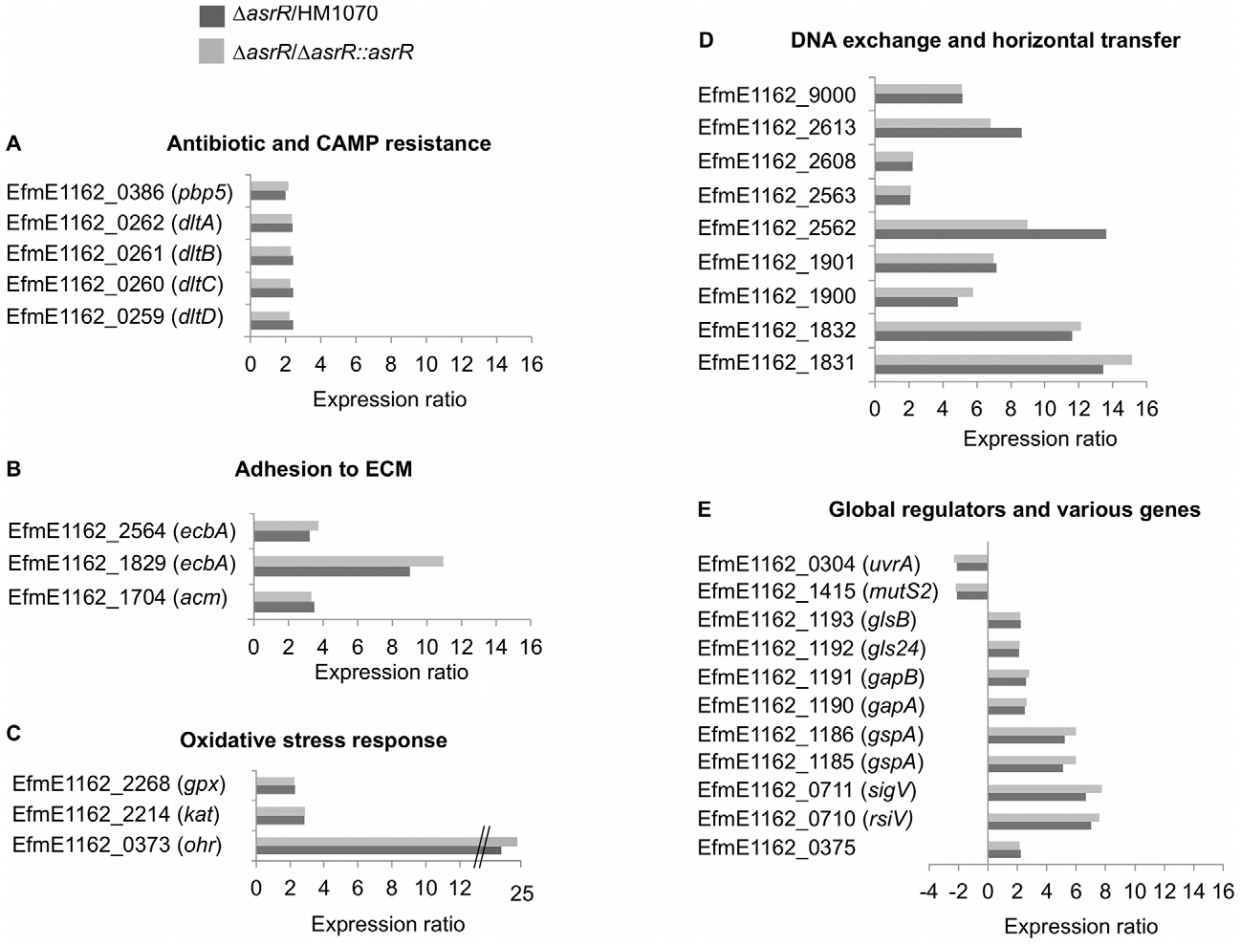
Key members of the AsrR regulon. (A) Members of the AsrR regulon involved in antibiotic and CAMP resistance, (B) adhesion to ECM, (C) oxidative stress response, (D) DNA exchange and horizontal transfer, and (E) global regulations pathways and various functions. Expression ratios for the mutant compared to the parental (Δ*asrR*/HM1070, black bar) or complemented (Δ*asrR*/Δ*asrR*::*asrR*, grey bar) strains are indicated. Gene tags or ORFs numbers are indicated following the E1162 strain annotation (Genbank accession ABQJ00000000) [7].

Both the parental strain and knock-in complemented derivatives were used for comparative transcriptome analysis to minimize the influence of unexpected random mutations that could have occurred during the construction of the *asrR* null mutant. We observed 87 genes significantly upregulated and 94 genes downregulated in the Δ*asrR* mutant strain in comparison to both the parental and complemented strains (Figure S3A). Nine and 33 genes showed modified transcriptionin the mutant when compared to the parent only or to the derivative only, respectively, and were not considered further (Figure S3A). To validate these results, we compared expression ratios obtained by microarrays and by qRT-PCR for seven selected genes and obtained an excellent correlation (r^2^ = 0.99) (Figure S3B). Expression ratios of key AsrR-regulated genes are shown in Figure 3.

Most genes shown in Figure 3 were up-regulated in the mutant and are classified in functional groups. A first functional group was composed of four genes homolog to those of the *dlt* operons involved in the resistance to cationic antimicrobial peptides (CAMPs) in *E. faecalis*, *Bacillus subtilis*, and *S. aureus* [29]–[31] as well as a *pbp5* gene, encoding a penicillin-binding protein responsible for β-lactam resistance in *E. faecium* (Figure 3A) [32]. Noticeably, genes involved in the adhesion to extracellular matrix (ECM) including the well-characterized *acm* gene [9], [33] and two *ecbA* paralogous genes [34] encoding microbial surface components recognizing adhesive matrix molecules (MSCRAMM) adhesins were also strongly up-regulated in the Δ*asrR* mutant (Figure 3B). Several genes putatively involved in oxidative stress response were up-regulated, including the *kat* and *gpx* genes encoding a putative manganese-containing catalase and a putative glutathione peroxidase, respectively, and the aforementioned *ohr* gene that was the most upregulated gene in the Δ*asrR* strain (Figure 3C). Numerous genes encoding putative transposon conjugative transfer proteins that could enhance DNA exchange and horizontal transfer were upregulated in the absence of AsrR (Figure 3D). In addition, homologs of genes known in other bacteria to be involved in the adaptation to environmental changes, *uvrA* and *mutS2* encoding an UV resistance determinant and a putative anti-recombination endonuclease, respectively, were downregulated in the Δ*asrR* strain (Figure 3E). *EfmE1162_0375* encoding a putative permease and located directly downstream of *asrR* was upregulated in the mutant as well as the *gspA* paralogous genes encoding general stress proteins (Figure 3E). Two glyceraldehyde-3-phosphate dehydrogenase (GAPDH) homologues of GapA and GapB, reported as *S. aureus* virulence factors [35], were upregulated in the Δ*asrR* strain (Figure 3E). The *gls24* and *glsB* genes, involved in bile salts stress response and virulence of *E. faecium*, were repressed by AsrR (Figure 3E) [36]. Finally, as previously described for several transcriptional regulators, *asrR* deletion also modulate expression of other transcriptional regulators, in particular that of SigV that was previously characterized in *E. faecalis* (Figure 3E) [30], [37].

Taken together, these results indicated that AsrR acts as a global regulator in *E. faecium*, functioning mainly as a repressor of numerous genes involved in antibiotic and CAMP resistance, adhesion to ECM, oxidative stress response and adaptative response. Using upstream regions of several of those genes upregulated in the *asrR* deleted mutant, we computationally identified a 15-bp putative DNA binding box (Figure S1D). In the following experiments, we tested the phenotypic effects of the modulation of expression of the various functional groups of genes.

### AsrR is involved in the oxidative stress response

**Figure 4:**
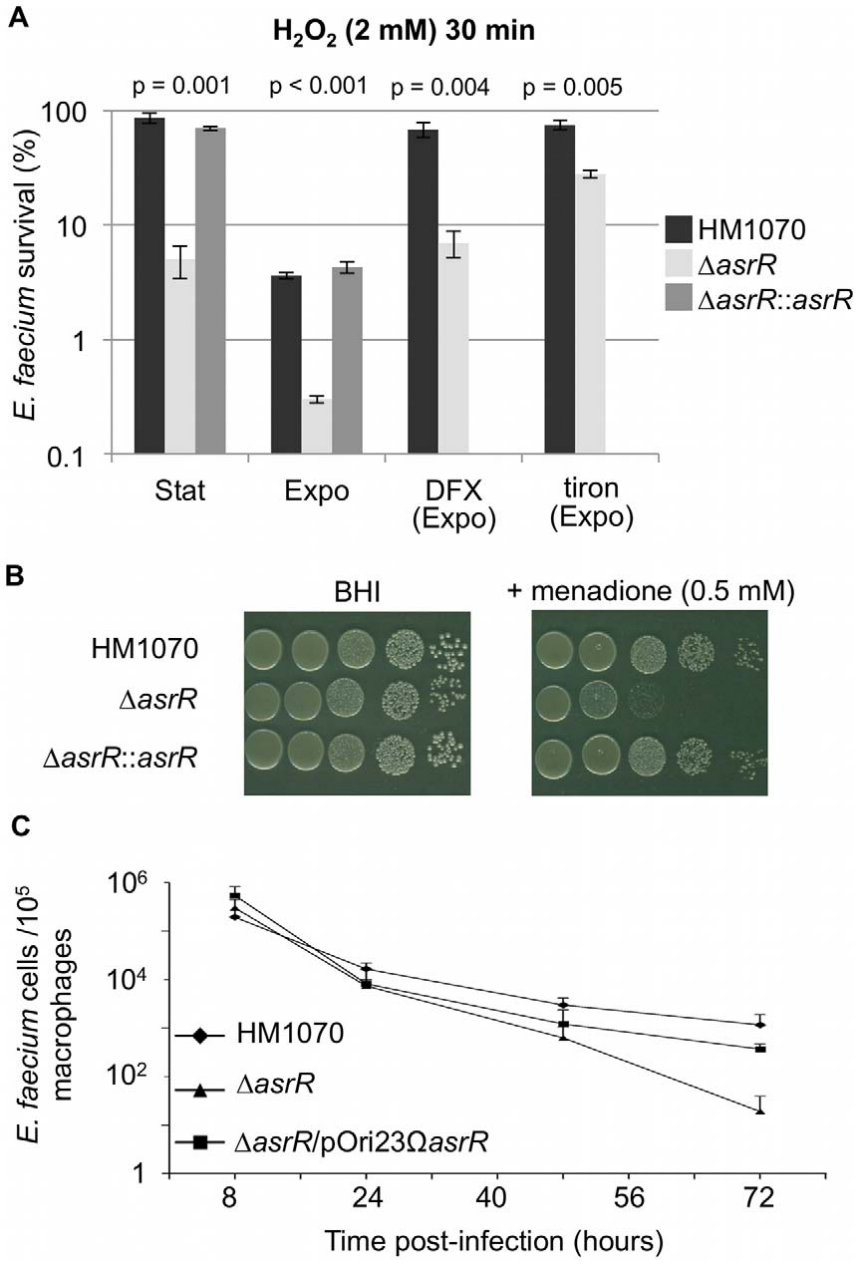
Role of AsrR in oxidative stress response. (A) Survival after a 30-min stress with 2 mM H_2_O_2_ for the parental (HM1070, black bar), mutant (Δ*asrR*, light grey bar) and complemented (Δ*asrR*::*asrR*, dark grey bar) strains. Cells were collected in stationnary phase (Stat), growth exponential phase (Expo), and exponential growth phase in the presence of deferoxamine (DFX) or tiron. Values are expressed as mean percentages (± standard deviation) of survival cells after oxidative stress compared to unstressed conditions from at least three independent experiments. (B) Menadione susceptibility of HM1070, Δ*asrR* and Δ*asrR*::*asrR* strains. *E. faecium* strains were 4-times serially diluted from a standardized (MacFarland = 1) cell suspension and spotted on BHI agar plates supplemented without or with (0.5 mM) menadione. Experiments were repeated at least three times and similar results were obtained. (C) Time course of intracellular survival of *E. faecium* parental (HM1070, diamonds), mutant (Δ*asrR*, triangles) and trans-complemented (Δ*asrR*/pOri23Ω*asrR*, squares) strains within murine peritoneal macrophages. Data are the mean numbers (± standard deviations) of viable intracellular bacteria per 10^5^ macrophages from three independent experiments in triplicate. The Δ*asrR* strain found to be more susceptible *in vitro* to H_2_O_2_ and menadione oxidative stress showed impaired survival in mouse macrophages compared to the parental and complemented strains.

*E. faecium* can survive a wide range of stresses during its life cycle. The role of AsrR in the response to H_2_O_2_ and organic oxidative stresses was tested by survival analysis and growth on plates containing oxidants (Figure 4A, Figure 4B). We performed survival experiments with a 2 mM H_2_O_2_ challenge for 30 min on cells in exponential or stationnary growth phases, and the Δ*asrR* strain was found to be around one order of magnitude more susceptible to hydrogen peroxide stress than the parental and complemented strains in both conditions (Figure 4A). Note that resistance to H_2_O_2_ dramatically decreased on growing cells (Figure 4A). We then performed a 2 mM H_2_O_2_ challenge for 30 min on cells in exponential growth phase in the presence of deferoxamine (DFX), an iron chelator, or tiron, a superoxide anion scavenger (Figure 4A). Interestingly, if the addition of DFX or tiron significantly increased the survival of both strains, as expected, the Δ*asrR* mutant was still significantly impaired as compared to the parental strain (Figure 4A). In addition, the growth on BHI plates of the Δ*asrR* derivative was also impaired by the addition of 0.5 mM menadione, an organic peroxide (Figure 4B). No significant differences were observed between the Δ*asrR* mutant and the parental strain when grown on plates containing other organic peroxides, such as tertiary-buthylhydroperoxide and cumene hydroperoxide (data not shown). Taken together, these results confirm that AsrR plays a role in the *E. faecium* oxidative stress response.

The role of AsrR in the oxidative stress response, was further tested *in vivo*. Survival of the parental, complemented and Δ*asrR* strains was monitored by counting of viable bacteria inside murine macrophages over a 3-day period (Figure 4C). Clearance of the Δ*asrR* mutant was slightly faster than that of the parent and of the complemented mutant, and correlated with its increased *in vitro* oxidative stress sensitivity.

**Figure 5:**
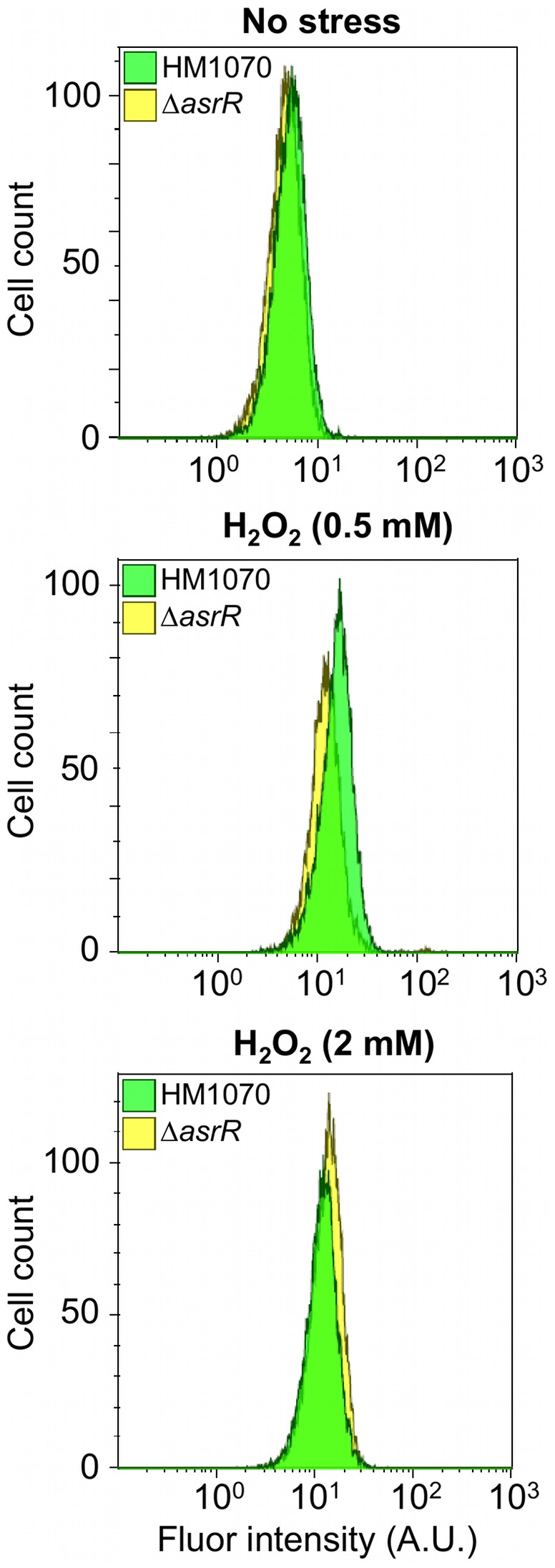
Measurement of intracellular hydroxyl radical concentration. The HM1070 (green) and Δ*asrR* (yellow) intracellular hydroxyl radical concentration was quantified by measuring the fluorescence intensity of a ROS-specific probe on cells under unstressed conditions or following a 10-min treatment with 0.5 mM or 2 mM of H_2_O_2_. Noticeably, if the intracellular hydroxyl radical concentration increases when exogenous H_2_O_2_ is added, no significant differences are found in intracellular ROS concentrations for the HM1070 and Δ*asrR* strains.

Finally, we estimated the intracellular concentration of hydroxyl radicals by FACS (fluorescence-activated cell sorting) experiments in both parental and Δ*asrR* mutant strains by measuring the fluorescence intensity of a probe specific for reactive oxygen species (ROS) (Figure 5). The basal hydroxyl radical level was similar in both strains (Figure 5). Interestingly, exogenous H_2_O_2_ treatment (0.5 mM or 2 mM, 10 min) increased the intracellular amount of hydroxyl radicals in both strains but no significant difference was found between the *E. faecium* HM1070 and Δ*asrR* strains (Figure 5).

### AsrR deletion promotes the resistance to antibiotics and CAMPs and protects from autolysis

**Figure 6:**
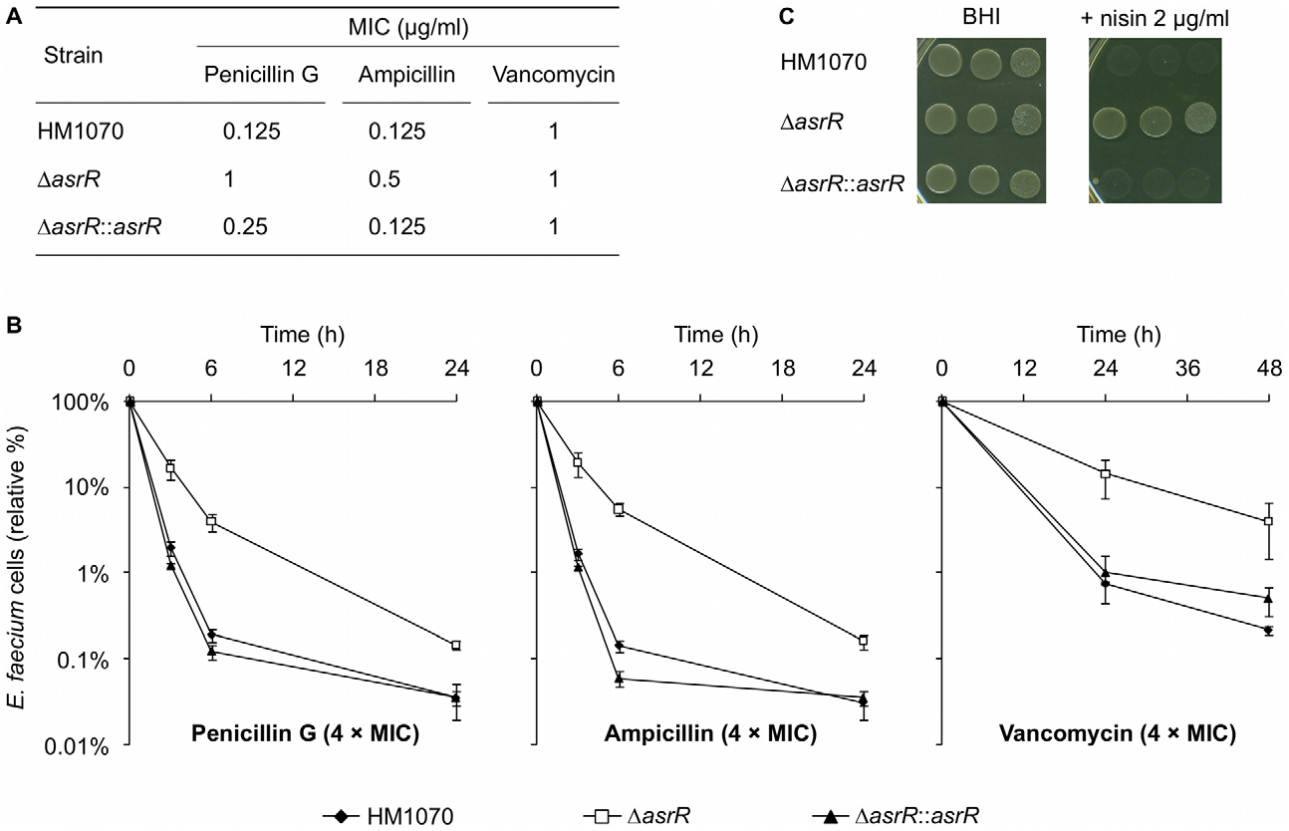
AsrR is associated with antibiotic and CAMP resistance. (A) MICs of penicillin G, ampicillin, and vancomycin for the parental (HM1070), mutant (Δ*asrR*) and complemented (Δ*asrR*::*asrR*) strains. Deletion of AsrR lead to decreased susceptibility of the mutant strain to β-lactams antibiotics. (B) Bactericidal activity of penicillin G, ampicillin, and vancomycin against HM1070 (black diamonds), Δ*asrR* (open squares), and Δ*asrR*::*asrR* (closed triangles) strains. For every strain, the antibiotic concentration was fixed at 4-fold of the MIC. Results are expressed as the percentage relative to the initial bacterial count. The Δ*asrR* strain was more resistant to the bactericidal activity of β-lactams and vancomycin compared to the parental and complemented strains. (C) Susceptibility to nisin of the parental (HM1070), mutant (Δ*asrR*), and complemented (Δ*asrR*::*asrR*) strains. *E faecium* strains were twice serially diluted from a standardized (MacFarland = 1) suspension and spotted on BHI agar plates without or with (2 µg/ml) nisin. Note that the Δ*asrR* strain was more resistant to nisin antimicrobial activity compared to parental and complemented strains. Experiments were repeated at least three times and similar results were obtained.

The effect of *asrR* deletion on the activity of various antimicrobials against *E. faecium* HM1070 was tested. The Δ*asrR* strain was more resistant to penicillin G and ampicillin (MIC of 1 and 0.5 µg/ml, respectively) than the parental (MIC of 0.125 µg/ml for both antibiotics) and the complemented strain (MIC of 0.25 and 0.125 µg/ml, respectively) (Figure 6A). These results are consistent with the *pbp5* gene upregulation in the Δ*asrR* mutant (Figure 3A). No differences were observed for vancomycin (MIC of 1 µg/ml for the three strains) (Figure 6A) and 24 other antibiotics tested (data not shown).

Because glycopeptides and β-lactams are bactericidal against *E. faecium*, we tested if *asrR* deletion could promote survival to these drugs. Time-kill analysis was carried out in the presence of vancomycin, penicillin G, and ampicillin (4× MIC). The bactericidal activity of penicillins and vancomycin against the Δ*asrR* strain was markedly reduced (by approximately one order of magnitude after 6 h and 24 h, respectively) as compared to the parental and complemented strains (Figure 6B). Finally, in agreement with tolerance to β-lactams [38], tests with Triton X-100 showed that autolysis was twice more rapid for the parental and the complemented strains than for the Δ*asrR* strain (Figure S4).

Bacterial cells have to cope with the CAMPs produced by other prokaryotic microorganisms and eukaryotic cells. The Δ*asrR* mutant exhibited noticeable growth on BHI plates supplemented with nisin (a bacterial CAMP) as compared to the parental and complemented strains (Figure 6C). No significant differences were observed between the Δ*asrR* mutant and the parental strain when grown on BHI plates containing colistin methanesulfate (data not shown). Previous studies have identified the *dlt* operon as crucial for response to CAMPs in numerous Gram-positive bacteria [29]–[31], [39] which is consistent with upregulation of *dlt* in the absence of AsrR.

### Lack of AsrR promotes the *E. faecium* biofilm formation and adhesion to epithelial cells

**Figure 7:**
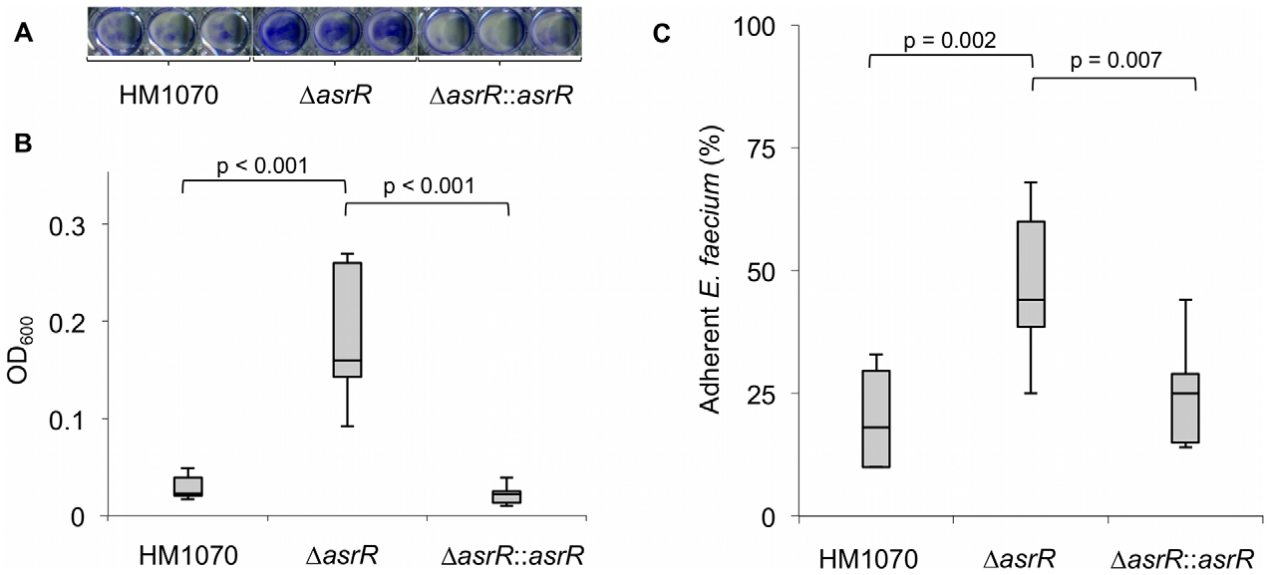
AsrR is involved in biofilm formation and adhesion to eukaryotic cells. (A) Direct observation of biofilm formation on polystyrene by the parental (HM1070), mutant (Δ*asrR*), and complemented (Δ*asrR*::*asrR*) strains, after a crystal-violet staining. (B) Ability of the strains to form biofilm on a polystyrene surface is shown after 24 h of incubation at 37°C. Values, measured with a microplate reader, are from three independent experiments performed in triplicate. Median and interquartile range values are shown. Note that the Δ*asrR* strain produced biofilm whereas parental and complemented strains did not produce biofilms under the tested conditions. (C) Adhesion of the strains to HT-29 cells. Adherence level is expressed as the percentage of adherent enterococci relative to the inoculum count. Values are from three independent experiments performed in triplicate. Median and interquartile range values are shown. The Δ*asrR* strain was found to be more adherent to epithelial HT-29 cells compared to parental and complemented strains.

Like other Gram-positive microorganisms, enterococci are able to produce biofilms on abiotic surfaces. The ability of Δ*asrR*, parental and complemented strains to form a biofilm on polystyrene microtiter plates was evaluated (Figure 7A). To quantify biofilm production, the OD_600_ in wells where bacteria have been cultured was determined after crystal-violet staining (Figure 7B). The parental and complemented strains did not produce biofilm after 24 h of incubation at 37°C whereas the *asrR* mutant adhered to the surface and formed significant amounts of biofilm (Figure 7B).

Adhesion to host-cells is a crucial step in the infection process and for host-colonization. Upregulation of the *acm* and *ecbA* genes, encoding major MSCRAMM adhesins, in the absence of AsrR prompted us to evaluate the contribution of AsrR to *E. faecium* adherence to HT-29 intestinal epithelial cells (Figure 7C). A high percentage of Δ*asrR* bacteria attached to the HT-29 cells (median value 44%), while the parental and complemented strains showed significantly lower levels of attachment (median values 18 and 25%, respectively) (*P*<0.01) (Figure 7C).

### AsrR deletion promotes the *E. faecium* mutagenesis and DNA transfer

Inactivation of the postreplicative DNA repair pathways has been shown in a wide variety of microorganisms to result in a mutator phenotype [40]. Since the *uvrA* gene, encoding a putative excision repair protein, was downregulated in the absence of AsrR, we determined the mutation frequency to spectinomycin resistance in the parent and the constructs. The Δ*asrR* strain displayed five-fold increase (*P* = 0.024) in mutation frequencies (4.4×10^−8^±1.8×10^−8^) as compared to the parental and complemented strains (respectively 8.5×10^−9^±1.1×10^−9^ and 9.1×10^−9^±1.3×10^−9^).

Considering both the strongly upregulated expression of genes involved in conjugation of transposons and the downregulated expression of *mutS2* in the absence of AsrR (Figure 3D and 3E), we studied the involvement of AsrR in DNA transfer. We conjugated the integrative conjugative transposon Tn*916* (which confers tetracycline resistance) from strain *Streptococcus agalactiae* UCN78 to *E. faecium* HM1070, Δ*asrR*, and Δ*asrR*::*asrR*, and subsequently from this set of strains to *E. faecalis* BM4110. Note that integration site of the Tn*916* in HM1070, Δ*asrR*, and Δ*asrR*::*asrR* strains did not influence the transfer frequency of three transconjugants tested for each constructed donor strains (data not shown). However, in three independent experiments, the Δ*asrR*/Tn*916* strains displayed a four-fold mean increase in Tn*916* transfer frequency (5.2×10^−6^±1.3×10^−6^) as compared to parental (1.5×10^−6^±2.2×10^−6^) and complemented (1.7×10^−6^±1.9×10^−6^) strains (*P* = 0.039).

### Deletion of *asrR* promotes the host colonization by *E. faecium*

**Figure 8:**
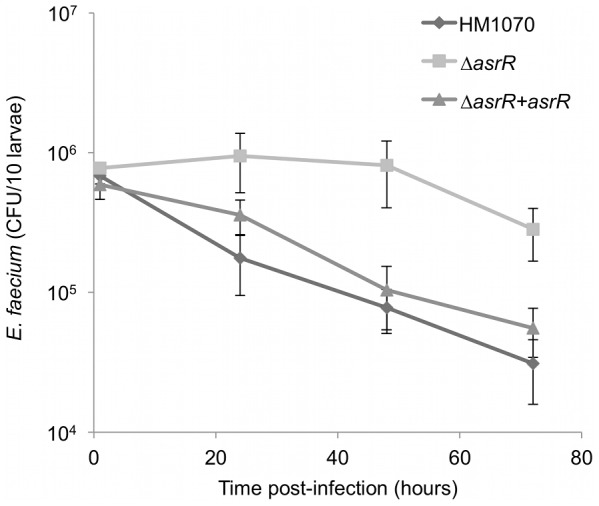
AsrR is associated with colonization of *G. mellonella* larvae. Loads of enterococci from *Galleria* larvae homogenates. Caterpillars were inoculated with 1.8×10^6^ (±0.5×10^6^) CFU of parental (HM1070, diamonds), mutant (Δ*asrR*, squares), and complemented (Δ*asrR*::*asrR*, triangles) strains. For each time point, homogenates of 10 alive larvae were plated for CFU count on selective agar plates. The Δ*asrR* strain was found to better colonize and persist in *Galleria* larvae compared to parental and complemented strains. Results represent means and standard deviations from at least three independent experiments.

Increased mammalian cell adhesion and biofilm formation of the Δ*asrR* mutant lead us to test the impact of AsrR on colonization of the host. To assay pathogenicity, we used larvae of the moth *Galleria mellonella* of which the innate immune system shares a high degree of structural and functional homology with that of mammals [41]. As described previously, only weak lethality for the larvae was observed with the parental strain [42] and no significant differences were found with the mutant (data not shown). Then, *Galleria* larvae were infected with Δ*asrR*, parental, or complemented strains, sacrificed at 0 h, 24 h, 48 h, and 72 h and bacterial counts were monitored in host homogenates (Figure 8). The parental and complemented loads markedly decreased following infection (from 1×10^6^ to 3×10^4^ and 5×10^4^ CFU/larva 72 h post-infection, respectively) whereas the Δ*asrR* load decreased only slightly, after stabilizing (2.8×10^5^ CFU/larva 72 h post-infection).

### Influence of AsrR on the *E. faecium* pathogenicity in a mouse systemic infection model

**Figure 9:**
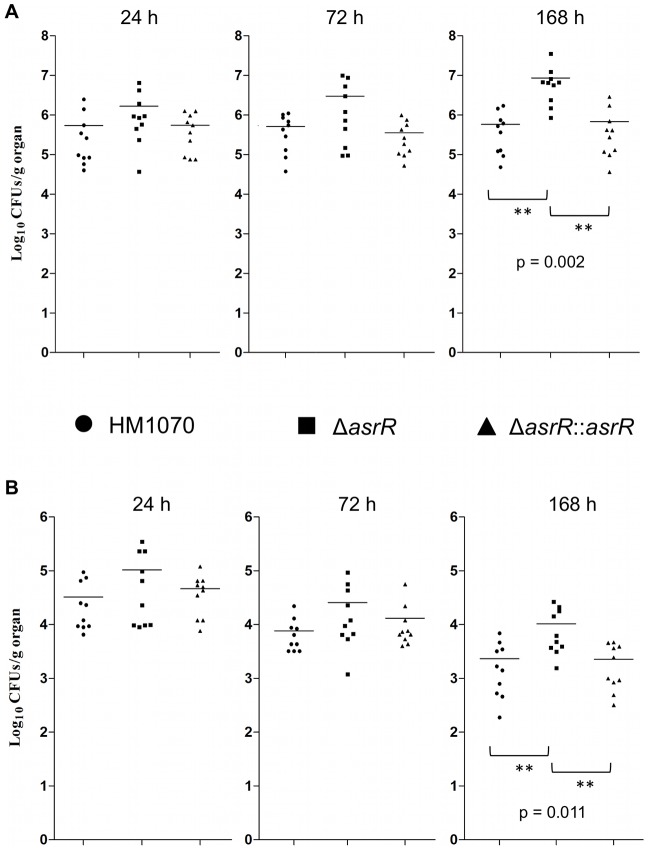
AsrR is associated with pathogenesis in a mouse intravenous infection model. Enterococcal tissue burdens in kidneys (A) and livers (B) of BALB/c mice infected intravenously with 1×10^9^ cells of the *E. faecium* HM1070 (circle), Δ*asrR* (square), and Δ*asrR*::*asrR* (triangle) strains. Groups of 10 mice were killed and necropsied 24 h, 72 h, and 168 h postinfection. The Δ*asrR* strain showed statistically significant increase of bacterial burdens in kidney and liver tissues at 168 h following injection, as compared to the parental and complemented strains. The results, expressed as log_10_ CFU per gram of tissue, represent the values recorded separately for each mouse. Horizontal bars represent the geometric means and significant differences are indicated (**).

In correlation with the insect model, the Δ*asrR* mutant strain showed statistically significant increase of bacterial burdens in kidney and liver tissues 168 h post-infection (Figure 9). The Δ*asrR* mutant exhibited an increase of 1.17 log unit in the kidneys (*P* = 0.002) (Figure 9A) and 0.70 log unit in the livers (*P* = 0.011) (Figure 9B) compared to the burdens of the HM1070 parent strain. The Δ*asrR::asr* complemented strain loads were restored to the wild-type level in both tissues confirming the involvement of AsrR in the *E. faecium* pathogenicity.

## Discussion

Reactive oxygen species were originally considered to be exclusively detrimental to bacterial cells. However, redox regulation involving ROS is now recognized as a vital component to bacterial signaling and regulation [43]–[45]. Some members of the MarR family modulate the transcription of virulence and/or stress genes using an oxidative sensing mechanism. In particular, studies on OspR of *P. aeruginosa* and MgrA of *S. aureus* have shown that the activity of these regulators that sense oxidative stress is not limited to oxidative stress response but has pleiotropic effects [15], [26]. The sensing mechanism of OspR has been recently described [26]. A cysteine residue, Cys-24, is used by OspR to sense a potential oxidative stress and to regulate bacterial response. Cys-24 is first likely oxidized and the resulting sulphenic intermediate is trapped by a second cysteine, Cys-134, to form an intermonomer disulphide bond. The inactive form of OspR dissociates from promoter DNA resulting in modulation of gene expression [26].

We identified a gene, *asrR*, which encodes a functional homologue of the OspR/MgrA proteins and is present in all sequenced *E. faecium* strains and absent in *E. faecalis*. Two cysteine residues are present in the protein sequence encoded by *asrR* indicating that AsrR belongs to the 2-Cys protein family, which senses peroxides [26], [46], [47]. Similarly to OspR, our data show that oxidative stress leads to inactivation of AsrR, resulting in loss of binding to promoter DNA, which leads to prompt modulation of gene expression.

Investigation of the AsrR regulon identified numerous targets consistent with the pleiotropic phenotype resulting from its inactivation. Oxidative stress acts as a signal modulating AsrR activity, but it remains a challenge to which bacteria have to cope with during infection. Indeed, our results show that ArsR played a role in the survival against H_2_O_2_ challenge as well as into phagocytic cells since three important genes from the oxidative stress regulon (i.e. *kat*, *gpx*, and *ohr*) are overexpressed in the absence of AsrR. However, this appears *a priori* in contradiction with the higher susceptiblity of the null-mutant strain to both *in vitro* oxidative stress and oxidative burst in mouse macrophages. Despite the fact that enterococci possess a *kat* gene, catalase enzyme can only be formed when heme or manganese is present and these organisms are considered as catalase-negative bacteria [48], [49]. While little is known about mechanisms of oxidative stress response in *E. faecium*, it has been shown in *E. faecalis* that peroxidases important for the survival under oxidative stress and into macrophages are Tpx (thiol peroxidase), Npr (NADH peroxidase), and Ahp (alkyl hydroperoxide reductase) [50], so that *E. faecium* homologs are not upregulated in the Δ*asrR* mutant. In *E. faecalis*, *gpx* encodes a glutathione peroxidase of which activity is regenerated by a glutathione reductase [51]. Since the gene encoding this reductase does not appear to be a member of the AsrR regulon, the impact of *gpx* overproduction alone on the oxidative damages restoration should be reduced in the mutant strain. Also, the data obtained with fluorescent ROS-specific probe confirmed that Δ*asrR* mutant strain did not better detoxify hydroxyl radicals than the wild-type strain. Interestingly, addition of ROS scavengers during the H_2_O_2_ challenge reduced the sensitivity of both wild-type and Δ*asrR* mutant strains, the mutant being still more sensitive. Under our conditions, it seems that hydrogen peroxide is capable of damaging the bacterial cell independently of the formation of hydroxyl radicals formed via the Fenton's reaction [52]. Therefore, although oxidative stress leads, through AsrR derepression, to overproduction of detoxification proteins, the absence of difference in intracellular ROS accumulation suggests that *asrR* deletion may lead to an increased oxidative susceptibility, independently of ROS detoxification pathways. One hypothesis is that a modification of the bacterial cell wall, for which evidence is also provided by our transcriptomic analysis and autolysis assay, may lead to increased oxidative susceptiblity [53].

AsrR regulation was not restricted to oxidative stress response but extended to modulation of expression of multiple targets. First, AsrR modulated resistance and tolerance to cell-wall active antimicrobial agents. In *E. faecium*, resistance to penicillins is due to production of the low-affinity penicillin-binding protein PBP5 [54] and overproduction of PBP5 increases the level of ampicillin resistance [32], [55]. Accordingly, the increase in MICs of penicillins after deletion of *asrR* in *E. faecium* HM1070 may be explained by upregulation of the *pbp5* gene. Interestingly, recent reports on the role of MgrA and OspR in antibiotic resistance reinforce the implication of these MarR regulators in β-lactam resistance [26], [56], [57]. In addition, the activity of the CAMP nisin, which damage the bacterial membrane, was reduced against the *asrR* null mutant. The *dlt* operon encodes proteins that alanylate teichoic acids, the major components of the cell wall of Gram-positive bacteria. This generates a net positive charge on bacterial cell walls, that repulses positively charged molecules and confers resistance to CAMPs [29], [31], [39]. Therefore, AsrR could contribute to modulate resistance to nisin in *E. faecium* through regulation of the *dlt* operon. These data suggest that the *E. faecium* Dlt resistance system is effective against CAMPs, as previously shown for *E. faecalis* [30].

Besides resistance, tolerance to antibiotics is an efficient pathway for bacteria to escape antimicrobial-induced killing. Bactericidal activity of both penicillins and vancomycin was significantly decreased in the *asrR* null mutant. Importantly, these antibiotics remain primary therapeutic choices for the treatment of enterococcal infections [58]. The molecular basis for tolerance remains poorly understood, and processes involved are much more complex than previously thought [59]. As suggested by transcriptomic data as well as biofilm and autolysis phenotypes, the higher tolerance to β-lactams and glycopeptides of the *asrR* mutant might be due to a modification of cell wall composition (peptidoglycan, lipoteichoic acids) and/or of intrinsic control of lysis (murein hydrolase activity) [59].

Similarly to other pathogens, adherence of *E. faecium* to exposed host ECM is likely the first step in the infection process. MSCRAMMs are proteins that adhere to components of the ECM [34]. To date, three MSCRAMMs, Acm, Scm and EcbA, have been characterized in *E. faecium* adhesion [34], [60]. Acm has been previously shown to interact with collagen type I and to a lesser extent with collagen type IV [33], [61], whereas Scm and EcbA bind to collagen type V and fibrinogen [60], [62]. The *in vivo* function of Acm has been thoroughly investigated highlighting its role in the pathogenesis of experimental *E. faecium* endocarditis [9]. Consistent with the literature, marked up-regulation of both *acm* and *ecbA* genes expression promotes ability of the null-mutant strain to adhere to epithelial cells. In addition, as previously described for MgrA in *S. aureus* [63], we report that AsrR represses biofilm formation in *E. faecium*. Following primary adhesion, biofilm establishes a protected environment for growth that enables bacteria to proliferate by restricting antibiotic access and shielding the bacterial pathogen from host immune defences [64].

Modulation by AsrR of biofilm formation and expression of MSCRAMM proteins indicates that this regulator may contribute to the host-colonization by *E. faecium*, a hypothesis confirmed in a *Galleria* persistence model and in a murine systemic infection model. *Galleria* insect model has been widely used to evaluate virulence of numerous pathogens [65]–[67] but has also recently been shown to be suitable for the study of *E. faecium* host-persistence [42]. Interestingly, the increased persistence of the null-mutant *E. faecium* strain inside larvae is correlated with its persistence following infection in mouse kidneys and liver. Good correlation between mouse and insect models has already been reported in the literature [68].

Beyond immediate cellular adaptation to stress, *E. faecium* organisms adapt their genome to hostile environmental conditions through acquisition of beneficial genes from external sources or by *de novo* mutations. The UV resistance genes (*uvr*) that are part of the SOS systems, have been analyzed in detail in *E. coli* and *E. faecalis* [40], [69], [70], and UvrA is the initial DNA damage-sensing protein in nucleotide excision repair [71]. AsrR deletion in *E. faecium* causes the downregulation of *uvrA* and, interestingly, the null mutant strain showed a mutator phenotype. We thus speculate that, under oxidative stress, *E. faecium* cells will promote mutations through AsrR-mediated deregulation of *uvrA* which would be globally profitable in hostile environments even though some may be deleterious to individual cells. Long-term adaptation may also benefit from genetic changes due to acquisition of pre-evolved functions via horizontal transfer [72]. Interestingly, we found that, in the absence of AsrR, *E. faecium* increases the transfer frequency of conjugative transposon Tn*916*, which might be linked to both the strong up-regulation of conjugative transposon protein and the down-regulation of the *mutS2* gene. Also, the DNA damage response role in the regulation of transfer of mobile genetic elements was previously described in *Bacillus subtilis* [73]. Considering the adaptive role of *uvr* and *mutS2* genes and DNA exchange, AsrR may contribute to the *E. faecium* long-term adaptation by modulating its mutability and DNA transfer capacities.

Lastly, the locus encoding the transcriptional regulator SigV, previously described as involved in *E. faecalis* virulence and CAMP response [30], was overexpressed in the mutant strain. It is likely that some members of the AsrR regulon are under several transcriptional controls. Currently, except for *ohr*, the possibility that AsrR modulates gene expression in *E. faecium* through interactions with other regulators cannot be excluded. Such a regulatory cascade has been shown for *E. faecalis*, *S. aureus*, and *Salmonella enterica* [13], [74], [75].

**Figure 10:**
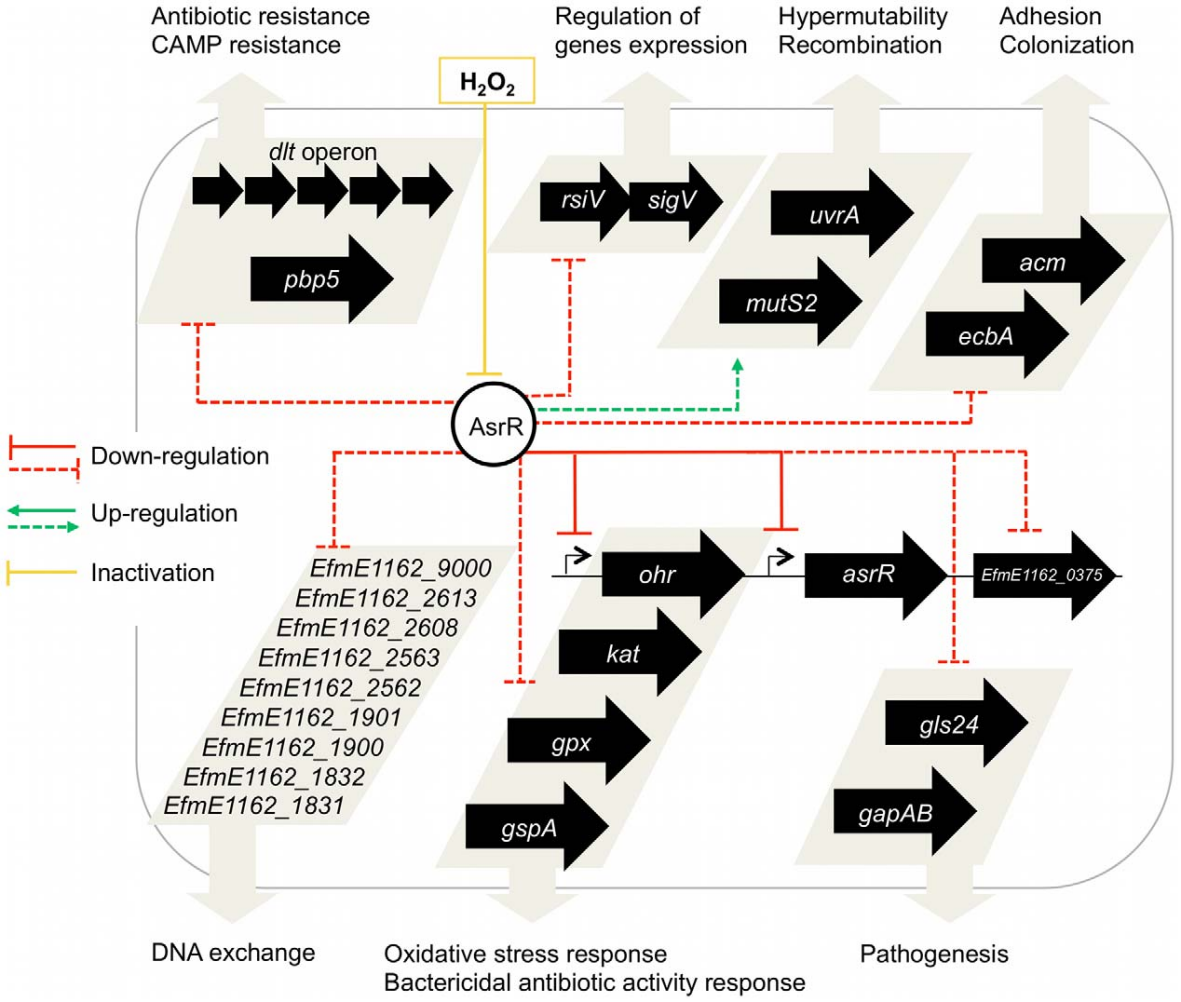
Proposed model for the role of AsrR in *E. faecium*. AsrR mediated up-regulations (green arrows) and down-regulations (red lines) and H_2_O_2_ inactivation of AsrR (yellow line) are indicated. Direct regulations demonstrated experimentally are represented as solid lines, whereas other regulations (direct or indirect) are represented as dashed lines. AsrR appears as a global repressor, inactivated by oxidative stress, of genes involved in important steps during the infection process.

The present investigation provides evidence that AsrR plays a key role in *E. faecium* adaptation, antimicrobial resistance and pathogenicity, which is summarized in a model (Figure 10). AsrR, which is inactivated in the presence of oxidative stress, is a global repressor (direct or indirect) of expression of the genes involved in all the important steps during the early infection process and allows host-colonization (Figure 10). It has been shown that nitric oxide-mediated activation of bacterial defence is important for the *in vivo* virulence of *Bacillus anthracis* [76]. Similarly, it can be speculated that the oxidative stress acts as a signal to promote the transition from the commensal to the opportunistic state thus rendering *E. faecium* more pathogenic.

## Materials and Methods

### Bacterial strains, plasmids and growth conditions

The strains and plasmids used in this study are listed (Table S1) [7], [77]–[80]. *E. faecium* was grown at 37°C in Brain Heart Infusion (BHI), Mueller-Hinton (MH) or Trypticase-Soy (TS) broth, or on BHI agar (Difco Laboratories). *Escherichia coli* were grown in Luria-Bertani (LB) broth or on LB agar (Difco Laboratories). When appropriate, antibiotics were added at the following concentrations: ampicillin 100 µg/ml; erythromycin 150 µg/ml for *E. coli* and 50 µg/ml for *E. faecium*; fusidic acid 40 µg/ml; kanamycin 30 µg/ml; lincomycin 30 µg/ml; rifampin 50 µg/ml; spectinomycin 150 µg/ml; streptomycin 150 µg/ml and tetracycline 100 µg/ml.

### DNA, RNA techniques and cloning

Plasmid pG(+)host9 is a temperature-sensitive *E. coli*–Gram-positive shuttle vector used for allelic replacement in Gram-positive bacteria [77]; pORI23 is an *E. coli*-Gram-positive shuttle vector used for complementation studies in *Lactococcus lactis* [78]. Vector pCR2.1-TOPO (Invitrogen) was used as recommended by the manufacturer for TA sub-cloning and cloning steps. Chromosomal DNA isolation, restriction endonuclease digestion, DNA ligation, and transformation of electrocompetent cells were performed using standard protocols or manufacturers instructions. For RACE-PCR and qRT-PCR, total *E. faecium* RNA was isolated using the RNeasy midi kit (Qiagen) as recommended by the manufacturer. For microarray experiments, total RNA was isolated as follows. Strains were cultured overnight in 3 ml of BHI broth within 10 ml tubes at 37°C with aeration by rotary shaking at 150 rpm and pre-warmed BHI broth (25 ml in 50-ml Falcon tube) was inoculated in duplicate with the overnight culture to a starting absorbance at 600 nm (OD_600_) of 0.025 and then incubated at 37°C as described above. One culture was used to monitor growth by measuring OD_600_. When OD_600_ reached 0.5 (mid-exponential growth phase), RNA was isolated from the second culture and the OD_600_ was measured to confirm equal growth in the duplicate cultures. For RNA isolation, 2 ml of each culture were transferred into an Eppendorf tube and spun down at 13,000×*g* for 20 s and the cell pellets were snap-frozen in liquid nitrogen. The time between removal from the incubator and freezing of the cell pellets was approximately 60 s. Within 20 min after freezing, 1 ml of TRI reagent (Ambion) was added to the frozen pellets and the suspension was transferred into a 2-ml tube filled with 0.5 g of 0.1 mm zirconia/silica beads (Biospec). Cells were disrupted by beadbeating three times for 1 min with intermittent cooling on ice. RNA was then extracted following Ambion's TRI reagent protocol. Residual chromosomal DNA was removed by treating samples with the TURBO DNA-*free* kit (Ambion). Extracted RNA samples were quantified using a Nanodrop 1000 spectrophotometer (Isogen Life Science) and stored in 70% ethanol-83 mM sodium acetate buffer (pH 5.2) at −80°C.

### PCR, Rapid Amplification of cDNA Ends (RACE-PCR), reverse transcriptase PCR (RT-PCR), and quantitative real-time PCR (qRT-PCR)

PCR amplification was carried out in a final volume of 50 µl containing 40 pmol of each oligonucleotide primer, *ca*. 100 ng of template DNA, using the GoTaq Flexi DNA polymerase kit (Promega) as recommended by the manufacturer. Primers used were designed based on the *E. faecium* HM1070 *asrR* cluster sequence (Accession number JQ390466) (Table S2) [81]. The transcriptional start sites of *asrR* and *ohr* were determined using the 5′ RACE system kit (Invitrogen) according to the manufacturer's instructions. The specific RACE-PCR primers were designed using the Primer3 software (http://frodo.wi.mit.edu/primer3) (Table S2).

For RT-PCR, cDNA was synthesized from total RNA (∼1.5 µg) by using the Superscript III First-Strand Synthesis System (Invitrogen, Breda, The Netherlands) according to the manufacturer's instructions. Using synthesized cDNAs, qRT-PCR was performed using Maxima SYBR Green/ROX qPCR Master Mix (Fermentas, St. Leon-Rot, Germany) and a StepOnePlus instrument (Applied Biosystems, Nieuwekerk a/d IJssel, The Netherlands) with the following program: 95°C for 10 min, and subsequently 40 cycles of 95°C for 15 sec, 55°C for 1 min. Relative transcript levels were calculated using REST 2009 Software (Qiagen). Expression of *tufA* was used as a housekeeping control gene.

### Construction of an HM1070 *asrR* deletion mutant and its complementation

The *asrR* deletion mutant (Δ*asrR*) was derived from *E. faecium* HM1070 by allelic exchange with a truncated copy of *asrR* as described [77]. Approximately 500 bp fragments upstream and downstream of *asrR* were amplified by PCR using HM1070 chromosome as template and primer pairs *asrR*-DC1-F/*asrR*-DC2-R and *asrR*-DC3-F/*asrR*-DC4-R (Table S2) [81]. The forward primer binding to the 3′-end of *asrR* (*asrR*-DC3-F) and the reverse primer to the 5′-end (*asrR*-DC2-R) were modified to carry the same restriction site (Table S2) [81]. Following restriction, ligation and amplification using *asrR*-DC1-F/*asrR*-DC4-R, the resulting fragment carrying the truncated *asrR* copy was cloned in the temperature-sensitive shuttle vector pG(+)host9 to create plasmid pG(+)host9Ω*asrR*-KO (Table S1) [7], [77]–[80]. The hybrid plasmid was introduced in the chromosome of HM1070 by electrotransformation and homologous recombination followed by excision of the wild-type copy as described [77]. In-frame deletion of the *asrR* gene was confirmed by PCR and sequencing. As described in Figure 1, around 42% of the sequence containing the helix-turn-helix DNA binding domain and the second Cys residue were deleted in the Δ*asrR* strain. No significant difference was found when comparing the growth kinetics of the parental and mutant strains (Figure S5). An *in trans* complemented Δ*asrR*/pOri23Ω*asrR* strain was constructed (Table S1) [7], [77]–[80], and complementation was confirmed in the *in vitro* physiological tests used in this study as well as in the murine macrophages experiments. Since only partial complementation was found with this construct (data not shown), we decided to construct the knock-in complemented strain, Δ*asrR*::*asrR* (Table S1) [7], [77]–[80]. Subsequently, all the experiments were conducted using the Δ*asrR*::*asrR* strain except for those with the mouse macrophages. For *asrR in trans* complementation, the *asrR* coding sequence from *E. faecium* HM1070 including the predicted ribosomal binding site was amplified with the primer pair *asrR*-pOri23-F/*asrR*-pOri23-R (Table S2) [81] and cloned in pOri23 (Table S1) [7], [77]–[80]. The resulting plasmid pOri23Ω*asrR* was introduced into the Δ*asrR* mutant strain by electrotransformation (Table S1) [7], [77]–[80]. For *asrR* knock-in complementation, the entire *asrR* sequence of HM1070 was amplified with the *asrR*-DC1-F/*asrR*-DC4-R primers (Table S2) [81] and cloned in pG(+)host9 to create plasmid pG(+)host9Ω*asrR*-KI which was introduced into the Δ*asrR* strain (Table S1) [7], [77]–[80], excised and allele replacement was obtained and verified as described above.

### Production of purified AsrR

A 460-bp fragment encoding AsrR was amplified by PCR from *E. faecium* HM1070 chromosome using primers AsrR-F and AsrR-R (Table S2) [81]. The product was cloned in pQE30 (Qiagen) downstream of the RGS-His_6_ tag sequence (Table S1) [7], [77]–[80]. The pQE30Ω*asrR* plasmid was electroporated in *E. coli* M15[pREP4] (Table S1) [7], [77]–[80], and expression of the his-tagged recombinant peptide was performed using IPTG induction (1 mM final concentration) as described [82]. Briefly, purification from *E. coli* M15[pREP4]/pQE30Ω*asrR* lysates was achieved by Ni^2+^-affinity chromatography using Ni-NTA resin (Qiagen) under native conditions. Samples were desalted on PD-10 columns (Amersham Biosciences) and protein concentrations were determined using the Bio-Rad protein assay.

### Electrophoretic mobility shift assays (EMSA) and footprinting experiments

DNA fragment from the *ohr* and *asrR* promoter regions was amplified, labelled by PCR with [γ-^32^P]dATP and incubated with purified His_6_-tagged AsrR (10 to 200 ng) in interaction buffer [40 mM Tris HCl [pH 7.5], bovine serum albumin 200 µg/ml, 2 mM CaCl_2_, 2 mM dithiothreitol and poly(dI-dC) µg/ml] at room temperature for 30 min. Designated amounts of H_2_O_2_ and or DTT were used as previously described [26]. The DNA-AsrR mixtures were electrophoresed in 12.5% polyacrylamide gels in 0.5× Tris-borate-EDTA (TBE) at 180 V that were dried and analyzed by autoradiography.

DNase I footprinting assays were performed as previously described [82] using a D-4 labelled DNA fragment of the *ohr* promoter. The capillary electrophoresis was performed using a CEQ8000 sequencing apparatus (Beckman Coulter). The determination of the DNA sequence of the protected region was performed after co-migration of the footprinting assay and the corresponding sequence reaction. The MEME suite (http://meme.sdsc.edu/meme/intro.html) was used to generate a putative AsrR binding box logo on several DNA sequences of regulon members.

### Genome sequence analysis

The *E. faecium* E1162 sequence was used as a reference and gene tags or ORFs numbers are indicated according to its annotation (Genbank accession ABQJ00000000) [7].

### cDNA synthesis, microarray design, and hybridization

Transcriptome comparisons were performed between the Δ*asrR* mutant, the parental HM1070 and the Δ*asrR*::*asrR* complemented strains grown to mid-exponential (OD_600_ = 0.5) phase. For each strain, bacterial RNA was extracted from four independent cultures as described above, and used for cDNA synthesis and labelling as detailed below. RNA samples were prepared and labelled with Cy3 or Cy5 as previsouly described [83]. Dyes were switched between samples to mimimize the effect of dye bias.

*E. faecium* microarrays (Agilent, Palo Alto, CA) were hybridized with 300 ng labelled cDNA. The experiments for comparison of the transcriptomes of the Δ*asrR* mutant, parental HM1070 or complemented Δ*asrR*::*asrR* strains were performed with four independent biological replicates. Slides were then scanned using an Agilent Technologies Scanner G2505B. Data were extracted from the scanned microarrays with Agilent Feature Extraction software (version 10.7.1), which includes a Lowess normalization step for the raw data. The microarrays used in this study were custom-made *E. faecium* E1162 arrays using Agilent's 8×15K platform (containing 8 microarrays/slide), as described previously [83].

### Analysis of microarray data

After removal of the data for the different controls printed on the microarray slides, the normalized data for each spot were analyzed for statistical significance using the Web-based VAMPIRE microarray suite (http://sasquatch.ucsd.edu/vampire/) [84], [85]. A spot was found to be differentially expressed between two samples using the threshold of a false discovery rate smaller than 0.05. An open reading frame was found to be differentially expressed when all four spots representing the open reading frame were significantly differentially expressed (False Discovery Rate for each spot <0.05) between samples. The average expression ratio of each significantly regulated open reading frame was determined by calculating the log-averages of the expression ratios of each individual probe. Finally, changes of 2-fold for upregulated and 0.5-fold for downregulated genes in the mutant strain were also introduced as biological significance limits.

### Microarray data and Genbank accession numbers

Microarray data were submitted to the MIAMExpress database and are accessible under accession number no. E-MEXP-3528. The nucleotide sequence of the *ohr*-*asrR* region in *E. faecium* HM1070 has been deposited in the GenBank database under accession no. JQ390466.

### Antibiotic susceptibility testing, determination of mutation frequencies and antibiotic time-kill analysis

Minimum inhibitory concentrations (MIC) were determined by the broth microdilution technique as recommended by the Comité de l'Antibiogramme de la Société Française de Microbiologie (http://www.sfm-microbiologie.org) [86]. For the determination of mutation frequencies, *ca.* 10^10^ cells from an overnight broth culture were plated onto BHI agar plates supplemented with spectinomycin and the mutation frequency was determined relative to the count of viable organisms plated in four independent experiments. Time–kill curves were determined for exponentially growing enterococcal cultures and an antibiotic concentration equal to 4× the MIC as described [87]. Briefly, bacteria were inoculated 1∶20 in 10 ml of fresh MH broth containing antibiotic and incubated at 37°C. Bacterial survival was monitored by CFU counts after 0, 3, 6, 24, and 48 h of incubation in three independent experiments by plating the cultures on BHI agar plates.

### Conjugation experiments

Transfer of Tn*916* carrying tetracycline resistance from strains *S. agalactiae* UCN78 (Table S1) [7], [77]–[80] to *E. faecium* HM1070, Δ*asrR* and Δ*asrR*::*asrR* was attempted by filter mating. Transconjugants were selected on BHI agar plates containing tetracycline, rifampicin and fusidic acid. For each strain, three transconjugants were selected and used in the following experiment to quantify the influence of the integration site on the transfer frequency. Transfer of Tn*916* from strains HM1070/Tn*916*, Δ*asrR*/Tn*916* and Δ*asrR*::*asrR*/Tn*916* to *E. faecalis* BM4110 was attempted as described above. Transconjugants were selected on BHI agar plates containing tetracycline, streptomycin and lincomycin whereas parental donor cells were selected on BHI agar plates containing rifampicin, fusidic acid and tetracycline. Transfer frequency data are of three independent experiments and statistical analysis was performed with the two-tailed Student's *t* test.

### Autolysis test

*E. faecium* Δ*asrR*, parental HM1070, and complemented strains grown in exponential growth phase in BHI were harvested, washed twice with cold phosphate-buffered saline (PBS; Gibco), resuspended in the same buffer supplemented with 0.1% Triton X-100 (Sigma), incubated at 37°C without shaking and autolysis was monitored by measuring the decrease in OD_600_ on a microplate reader system (Multiskan Ascent, Thermo Electron Corporation). The initial OD_600_ value was fixed at 100%, and the results are the means (± standard deviation) from three independent experiments.

### Susceptibility to CAMPs

*E faecium* HM1070, Δ*asrR* mutant and complemented strains suspensions were standardized to an OD_600_ of 1 in 0.9% NaCl and 10 µl aliquots of 10-fold dilutions were spotted on BHI agar plates supplemented with various amounts of colistin methanesulfate (Sigma) and nisin (Sigma). Experiments were repeated at least three times and representative data are shown.

### Oxidative stress

*E faecium* HM1070, Δ*asrR* mutant, and complemented strains suspensions were standardized to an OD_600_ of 1 in 0.9% NaCl and 10 µl aliquots of 10-fold dilutions were spotted on BHI agar plates supplemented with various amounts of menadione, tertiary-buthylhydroperoxide, and cumene hydroperoxide. Experiments were repeated at least three times and representative data are shown.

### H_2_O_2_ killing assays

Resistance of *E. faecium* to oxidative killing by H_2_O_2_ was tested as described with slight modifications [88]. Bacteria were grown 16 h in BHI broth and sub-cultured in 10 ml broth at a starting density of OD_600_ at 0.05. Cultures were grown to mid-exponential phase (OD_600_ = 0.5) or to stationary phase (OD_600_ = 1.4), harvested by centrifugation, resuspended in 0.9% NaCl with 2 mM H_2_O_2_, placed into a 37°C water bath, and samples were enumerated on plates immediately and 30 min following H_2_O_2_ challenge. For H_2_O_2_ killing assays in the presence of iron or superoxide anion scavengers, cultures (OD_600_ = 0.5) were resuspended in 2 mM H_2_O_2_-containing 0.9% NaCl supplemented with 100 µM of deferoxamine or 3.3 mM tiron, respectively, and processed as described [88].

### Measurement of hydroxyl radical concentration using FACS

The detection of intracellular hydroxyl radical was carried out as described [89]. All data were collected using a Epics XL Beckman Coulter flow cytometer with a 488 nm argon laser and a 505–545 nm emission filter (FL1) at low flow rate. In all experiments, cells were grown as described above, stressed with 0, 0.5, or 2 mM of H_2_O_2_ during 10 min and washed with PBS buffer. At least 30,000 cells were collected for each sample. To detect hydroxyl radical formation, we used the fluorescent reporter dye 3′-(p-hydroxyphenyl) fluorescein (HPF; Invitrogen) at a concentration of 10 µM. Flow data were processed and analyzed with Kaluza V1.2.

### Biofilm formation

Bacteria that had been grown overnight were inoculated 1∶100 in 10 ml of TS broth with 0.25% glucose and shared into 96-microwell polystyrene plates (NUNC, Denmark). After 24 h of static incubation at 37°C, the plates were washed three times with PBS and stained with 1% crystal violet for 30 min. The wells were rinsed with distilled water and ethanol-acetone (80∶20, vol/vol). After drying, OD_600_ was determined using a microplate reader (Multiskan Ascent, Thermo Electron Corporation). Each assay was performed in triplicate in at least three independent experiments. For visualization, bacteria were grown as described above using 12-well polystyrene plates (CytoOne, Starlab International, Germany) and were similarly processed than above and directly examined.

#### *E. faecium* adhesion assays

Human cell line HT-29, derived from colon adenocarcinoma, was used to assess *E. faecium* adhesion ability. Cells were cultivated in Dulbecco's Modified Eagle Medium (DMEM) (Lonza, Verviers, Belgium) supplemented with 10% heat-inactivated fetal bovine serum (PAA, Pasching, Austria), 1% L-glutamine (Gibco, Paisley, UK), and 1% penicillin-streptomycin liquid (Gibco). Cells were seeded at 1×10^6^ cells in 10 ml DMEM in 25 cm^2^ culture bottles (Greiner bio-one, Frickenhausen, Germany) and incubated at 37°C with 5% CO_2_. Experiments were performed on cells after 10–15 passages. HT-29 cells were collected every 4–5 days by washing the monolayer with PBS and trypsinizing the cells with 50 µg/ml trypsine (Gibco). Cells were seeded in 12-well tissue culture plates (CytoOne, Starlab International) at ∼2×10^5^ cells/ml. HT-29 cells were used 7 to 10 days after seeding. Overnight bacterial cultures in TS broth supplemented with 0.25% glucose were diluted (1∶50) and grown at 37°C to an OD_600_ of 0.5, harvested by centrifugation and resuspended in DMEM (1×10^7^ CFU/ml). Wells with HT-29 monolayer cells were rinsed with DMEM and 1 ml of bacterial suspension was inoculated (ratio of 100 bacteria to 1 epithelial cell). Plates were centrifuged, incubated with bacteria for 2 h at 37°C in 5% CO_2_, monolayers were rinsed three times with DMEM and cells were lysed with 1% Triton X-100 (Sigma-Aldrich, USA). As a negative control, gentamicin (20 µg/ml) was added and cells were incubated 1 h at 37°C in 5% CO_2_ prior rinsing. Adherent bacteria were plated on BHI agar plates and quantified by CFU counting. The assay was performed in triplicate (3 wells per strain) and repeated three independent times.

### *Galleria mellonella* colonization model

The *in vivo* colonization model was carried out as described [42]. *Galleria* larvae were infected (1.8×10^6^±0.5×10^6^ CFU/larva) and batches of 10 alive larvae were sacrificed at 0, 24, 48, and 72 h post-infection and homogenized as described previously [42]. The t_0_ time point was determined immediately following injection. Homogenates were plated onto BHI agar plates containing aztreonam (100 µg/ml) and rifampicin (60 µg/ml), and CFU were counted after 24 h of incubation. Results represent means (± standard deviation) of at least three independent experiments.

### Assays of survival in murine peritoneal macrophages and in a mouse systemic infection model

Mouse experiments were performed with the approval of an institutional animal use committee (see below). Mice were housed in filter-top cages and had free access to food and water.

The *in vivo-in vitro* model of survival within murine macrophages was carried out as described [90]. Briefly, *E. faecium* strains were grown in BHI for 16 h, pelleted and resuspended in an adequate volume of PBS for injection. Male BALB/c mice (10 weeks old; Harlan Italy S.r.l.) were infected with 10^7^ to 10^8^ cells by intraperitoneal injection and after 6 h infection macrophages were collected by peritoneal lavage, centrifuged, and suspended in DMEM containing 10 mM HEPES, 2 mM glutamine, 10% bovine fetal serum, and 1× nonessential amino acids supplemented with vancomycin (10 µg/ml) and gentamicin (150 µg/ml). The cell suspension was dispensed into 24-well tissue culture plates and incubated at 37°C under 5% CO_2_ for 2 h. After exposure to antibiotics to kill extracellular bacteria (i.e., at 8 h postinfection), the infected macrophages were washed, and triplicate wells of macrophages were lysed with 0.1% sarkosyl. Note that, nor the HM1070 strain, neither the Δ*asrR* mutant were found to be sensitive to the lytic treatment (data not shown). The lysates were diluted in BHI broth and plated on BHI agar to quantify the number of viable intracellular bacteria. The remaining wells were maintained in DMEM with the antibiotics for the duration of the experiment. The same procedure was performed at 24, 48, and 72 h post-infection. All experiments were performed at least three times.

The intravenous systemic infection model was performed as described previously [91]. Briefly, overnight cultures of the strains grown in BHI broth supplemented with 40% heat-inactivated horse serum were centrifuged, and the resulting pellets were resuspended in sterile PBS to achieve final concentrations of 1×10^9^ cells/ml. Aliquots of 100 µl from each strain suspension were used to inject the tail veins of groups of 10 mice each. The infection experiments were repeated three times. The mice were monitored with twice-daily inspections, and 1, 3, and 7 days after infection they were killed using CO_2_ inhalation. The kidneys and livers were then removed aseptically, weighed, and homogenized in 5 ml of PBS for 120 s at high speed in a Stomacher 80 apparatus (Pbi International). Serial homogenate dilutions were plated onto *Enterococcus* Selective Agar (ESA; Fluka Analytical) to determinate the CFU numbers. All experiments were performed three times.

### Ethics statement

The mouse experiments were performed under a protocol approved by the Institutional Animal Use and Care Committee at the Università Cattolica del Sacro Cuore, Rome, Italy (Permit number: Z21, 11/01/2010) and authorized by the Italian Ministry of Health, according to the Legislative Decree 116/92, which implemented the European Directive 86/609/EEC on laboratory animal protection in Italy. Animal welfare was routinely checked by veterinarians of the Service for Animal Welfare.

### Statistical analysis

Comparisons between groups were performed with different statistical tests (one-way analysis of variance with a Bonferroni correction post test or non-parametric Wilocoxon signed-rank sum test) using GraphPad Prism software (version 5.00) for Windows (GraphPad Software, San Diego, CA). For all comparisons, a *P* value of less than 0.05 was considered as significant.

## Supporting Information

Figure S1 **Analysis of the transcriptional unit encoding** ***ohr*** **and** ***asrR*** **genes.** (A) The cotranscription of *asrR* from *ohr* promoter was evaluated by RT-PCR on total RNA from HM1070 using primers designed to amplify specific region of *ohr* (1) or *asrR* (3), intergenic region (2), the long cotranscript (4) and a negative control (c) (Table S2) [81]. (B) Agarose gel showing the corresponding PCR products. Lanes c and c′ represent PCR amplifications on chromosomal DNA or on cDNA, respectively, used as controls. (C) Footprinting experiment performed on the *ohr* promoter in the absence (red line) or presence (blue line) of the His_6_-tagged AsrR purified protein. The corresponding *ohr* promoter sequence is showed and the binding region is indicated in bold characters. (D) Alignment of the AsrR binding site for five putative direct target genes. The distance of the last nucleotide of the 15-bp binding sites to the start codons is indicated (*Location*). The DNA sequence logo representing the AsrR DNA binding site in *E. faecium* was created using the MEME suite and represents the information content of the alignment of AsrR DNA binding sites, showing the sequence conservation (overall height at each position) and the relative frequency of each nucleotide at each position (nucleotide height).
(TIF)

Figure S2 **Oxidative stress modulates expression of both** ***ohr*** **and** ***asrR*** **genes.** Expression ratios of the *asrR* (grey bar) and *ohr* (black bar) genes determined by qRT-PCR at different times following a 2 mM H_2_O_2_ stress. Expression ratios for the parental strain in the presence of H_2_O_2_ compared to the parental strain without oxidative stress (HM1070+ H_2_O_2_/HM1070) are indicated. Note that the *ohr* and *asrR* genes are rapidly and strongly upregulated in the presence of H_2_O_2_.
(TIF)

Figure S3 **qRT-PCR confirms the AsrR regulon identified by microarrays.** (A) Comparison of differentially expressed genes between Δ*asrR* mutant and wild-type (Δ*asrR*/HM1070 ratios) or complemented (Δ*asrR*/Δ*asrR*::*asrR* ratios) strains. The number of genes differentially expressed between the mutant (Δ*asrR*) and the parental (HM1070) or complemented (Δ*asrR*::*asrR*) strains are indicated in the circles. The black overlapping area indicates that the genes differentially expressed in the mutant compared to both the parent and the complemented derivative. (B) Correlation of microarrays and qRT-PCR expression ratios for the seven gene members of the AsrR regulon. Genes expression for *uvrA* (orange symbols), *pbp5* (blue symbols), *gpx* (green symbols), *kat* (purple symbols), *acm* (red symbols), *sigV* (grey symbols), and *ohr* (brown symbols) are indicated. Expression ratios *asrR*/HM1070 (circles, blue correlation line) and *asrR*/Δ*asrR*::*asrR* (squares, red correlation line) are indicated.
(TIF)

Figure S4 **Impact of** ***asrR*** **deletion on the** ***E. faecium*** **autolysis.** Autolysis of the parental (HM1070, diamonds), mutant (Δ*asrR*, squares), and complemented (Δ*asrR*::*asrR*, triangles) strains. Autolysis was induced by addition of Triton X-100 (0.1%) and monitored using a microplate reader. Note that autolysis rate was higher in the parental and the complemented strains than in the Δ*asrR* strain. Results, expressed as percentage of initial OD_600_, are from three independent experiments.
(TIF)

Figure S5 **Growth curves of the parental HM1070 and the Δ** ***asrR*** **mutant strains.** Growth for the parental *E. faecium* HM1070 (black squares) and the Δ*asrR* mutant (grey circles) were carried out in BHI at 37°C and monitored every 30 min. No significant difference was observed between the two strains.
(TIF)

Table S1 **Plasmids and strains used in this study.**
(DOC)

Table S2 **Oligonucleotide primers used in this study.**
(DOC)
