# Supplementary figures and images for: AsrR Is an Oxidative Stress Sensing Regulator Modulating Enterococcus faecium Opportunistic Traits, Antimicrobial Resistance, and Pathogenicity
Source: PLoS Pathog. 2012 Aug 2;8(8):e1002834. doi: 10.1371/journal.ppat.1002834 (PMC3410868; doi:10.1371/journal.ppat.1002834)

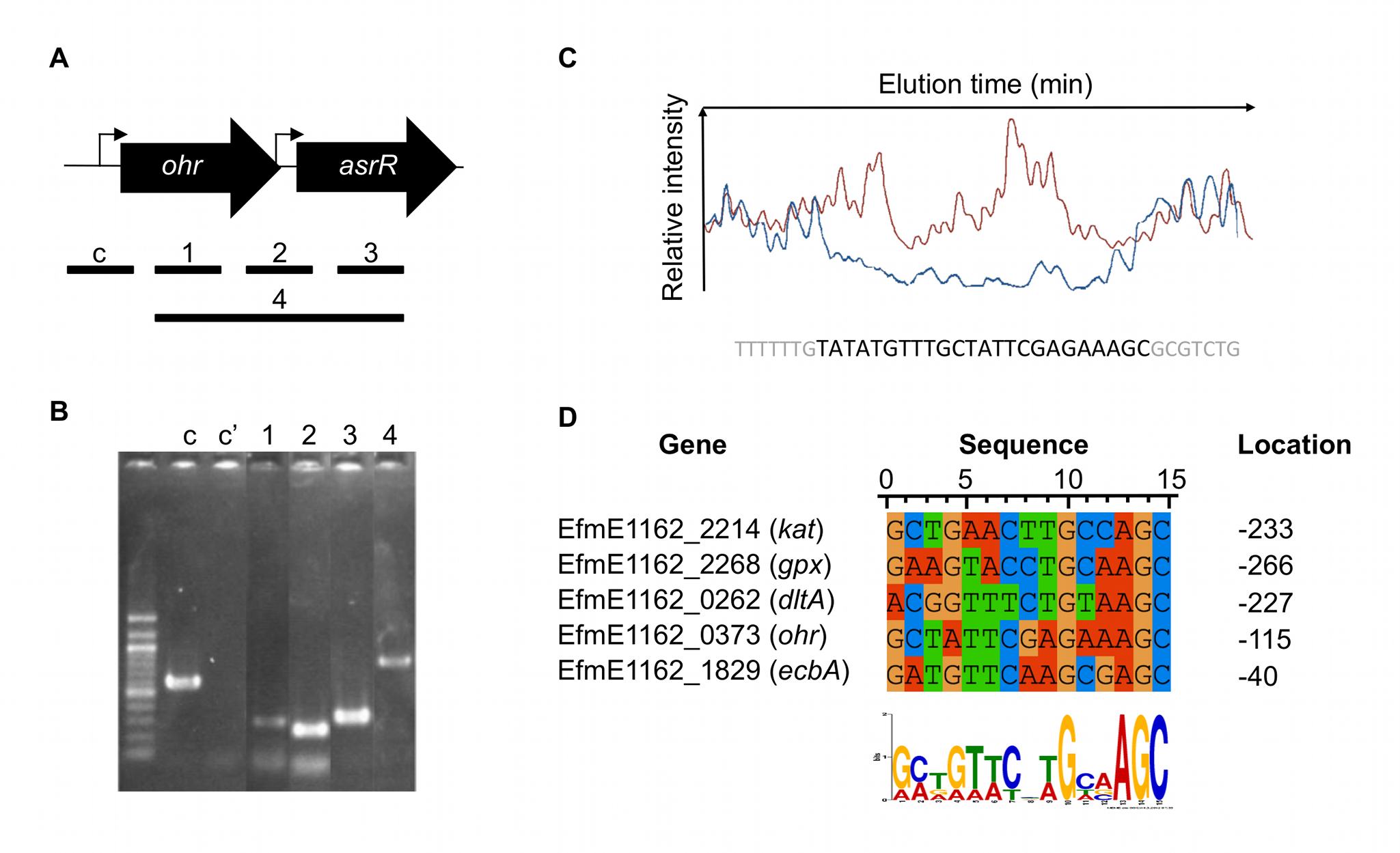

Supplement: Figure S1 — Analysis of the transcriptional unit encoding ohr and asrR genes. (A) The cotranscription of asrR from ohr promoter was evaluated by RT-PCR on total RNA from HM1070 using primers designed to amplify specific region of ohr (1) or asrR (3), intergenic region (2), the long cotranscript (4) and a negative control (c) (Table S2) [81]. (B) Agarose gel showing the corresponding PCR products. Lanes c and c′ represent PCR amplifications on chromosomal DNA or on cDNA, respectively, used as controls. (C) Footprinting experiment performed on the ohr promoter in the absence (red line) or presence (blue line) of the His6-tagged AsrR purified protein. The corresponding ohr promoter sequence is showed and the binding region is indicated in bold characters. (D) Alignment of the AsrR binding site for five putative direct target genes. The distance of the last nucleotide of the 15-bp binding sites to the start codons is indicated (Location). The DNA sequence logo representing the AsrR DNA binding site in E. faecium was created using the MEME suite and represents the information content of the alignment of AsrR DNA binding sites, showing the sequence conservation (overall height at each position) and the relative frequency of each nucleotide at each position (nucleotide height). (TIF) [file ppat.1002834.s001.tif]

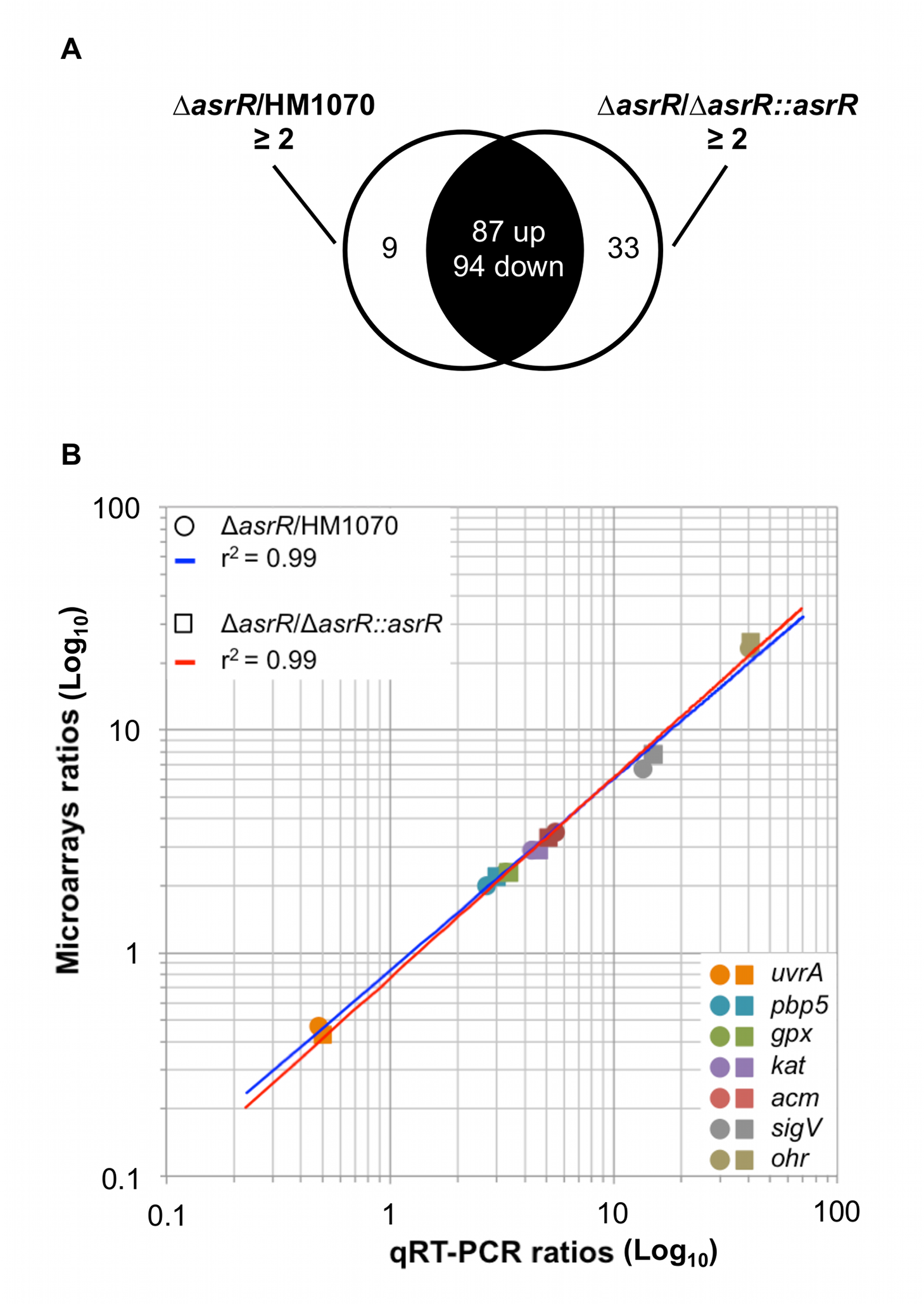

Supplement: Figure S3 — qRT-PCR confirms the AsrR regulon identified by microarrays. (A) Comparison of differentially expressed genes between ΔasrR mutant and wild-type (ΔasrR/HM1070 ratios) or complemented (ΔasrR/ΔasrR::asrR ratios) strains. The number of genes differentially expressed between the mutant (ΔasrR) and the parental (HM1070) or complemented (ΔasrR::asrR) strains are indicated in the circles. The black overlapping area indicates that the genes differentially expressed in the mutant compared to both the parent and the complemented derivative. (B) Correlation of microarrays and qRT-PCR expression ratios for the seven gene members of the AsrR regulon. Genes expression for uvrA (orange symbols), pbp5 (blue symbols), gpx (green symbols), kat (purple symbols), acm (red symbols), sigV (grey symbols), and ohr (brown symbols) are indicated. Expression ratios asrR/HM1070 (circles, blue correlation line) and asrR/ΔasrR::asrR (squares, red correlation line) are indicated. (TIF) [file ppat.1002834.s003.tif]

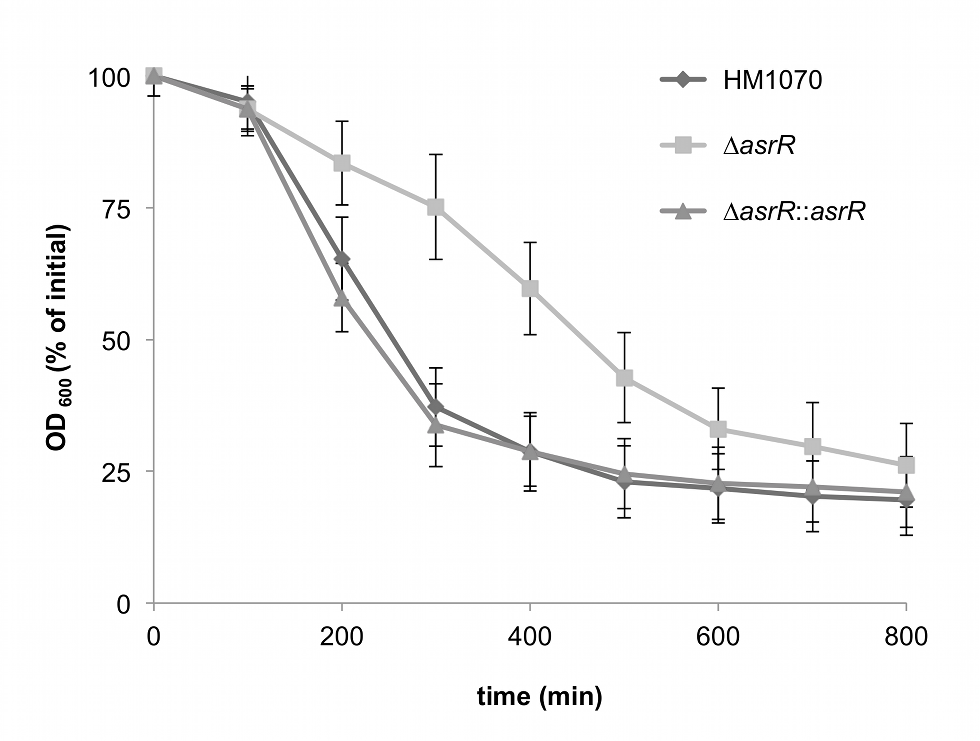

Supplement: Figure S4 — Impact of asrR deletion on the E. faecium autolysis. Autolysis of the parental (HM1070, diamonds), mutant (ΔasrR, squares), and complemented (ΔasrR::asrR, triangles) strains. Autolysis was induced by addition of Triton X-100 (0.1%) and monitored using a microplate reader. Note that autolysis rate was higher in the parental and the complemented strains than in the ΔasrR strain. Results, expressed as percentage of initial OD600, are from three independent experiments. (TIF) [file ppat.1002834.s004.tif]

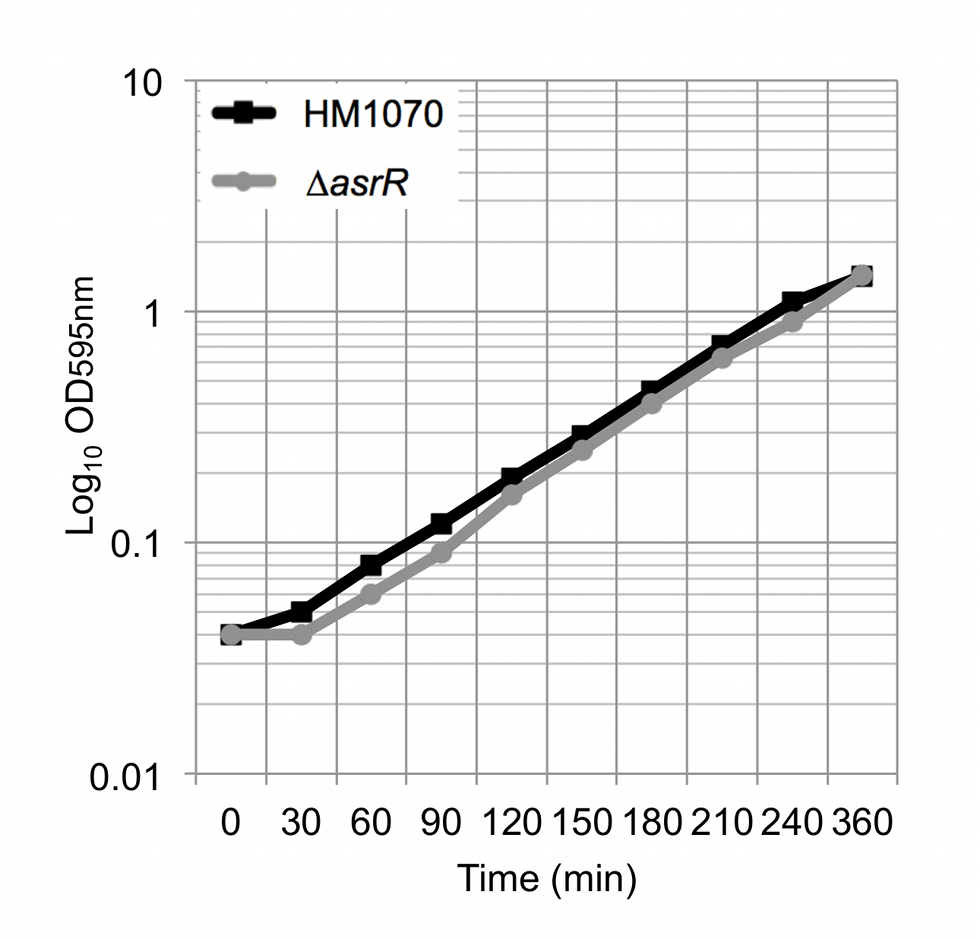

Supplement: Figure S5 — Growth curves of the parental HM1070 and the Δ asrR mutant strains. Growth for the parental E. faecium HM1070 (black squares) and the ΔasrR mutant (grey circles) were carried out in BHI at 37°C and monitored every 30 min. No significant difference was observed between the two strains. (TIF) [file ppat.1002834.s005.tif]
