# Supplementary material for: AsrR Is an Oxidative Stress Sensing Regulator Modulating Enterococcus faecium Opportunistic Traits, Antimicrobial Resistance, and Pathogenicity
Source: PLoS Pathog. 2012 Aug 2;8(8):e1002834. doi: 10.1371/journal.ppat.1002834 (PMC3410868; doi:10.1371/journal.ppat.1002834)
Supplement: Table S1 — Plasmids and strains used in this study. (DOC) [file ppat.1002834.s006.doc]

# Table S1. Plasmids and strains used in this study.

| **Plasmids or strains** | **Relevant characteristics and antibiotic resistances** | **Source** |
| --- | --- | --- |
| **Plasmids** |  |  |
| **pG(+)host9** | *ori*pWV01, thermosensitive replication vector used for mutagenesis, Ery | [77] |
| **pOri23** | *ermAM*, *ori*ColE1, P23, Ery | [78] |
| **pREP4** | Plasmid containing the *lacI* gene, compatible with pQE30, Kan | Qiagen |
| **pQE30** | IPTG inducible expression, N-terminal His tag, Amp | Qiagen |
| **pCR2.1-TOPO** | General AT-cloning vector | Invitrogen |
| **pG(+)host9Ω*asrR*-KO** | pG(+)host9 derivative carrying *asrR*-KO for the targeted *asrR* knock-out | This study |
| **pG(+)host9Ω*asrR*-KI** | pG(+)host9 derivative carrying *asrR*-KI for the targeted asrR knock-in | This study |
| **pOri23Ω*asrR*** | pOri23 derivative carrying *asrR* for trans-complementation | This study |
| **pQE30Ω*asrR*** | pQE30 derivative carrying *asrR* for protein expression and purification | This study |
| **pCR2.1Ω*ohr-prom*** | pCR2.1-TOPO derivative carrying the *ohr* promoter | This study |
|  |  |  |
| **Strains** |  |  |
| ***Enterococcus faecium*** |  |  |
| **E1162** | Microarray-based, sequenced and annotated reference strain | [7] |
| **HM1070** | Wild-type, Fus, Rif | [79] |
| **∆*asrR*** | HM1070 derivative with an in-frame deletion of *asrR* | This study |
| **∆*asrR/*pOri23Ω*asrR*** | ∆*asrR* trans-complemented strain carrying pOri23Ω*asrR,* Fus, Rif, Ery | This study |
| **∆*asrR*::*asrR*** | ∆*asrR* knock-in complemented strain | This study |
| **HM1070/Tn*916*** | HM1070 carrying Tn*916* (3 strains #1, #2 and #3), Fus, Rif, Tet | This study |
| **∆*asrR*/Tn*916*** | ∆*asrR* carrying Tn*916* (3 strains #1, #2 and #3), Fus, Rif, Tet | This study |
| **∆*asrR*::*asrR*/Tn*916*** | ∆*asrR*::*asrR* carrying Tn*916* (3 strains #1, #2 and #3), Fus, Rif, Tet | This study |
|  |  |  |
| ***Enterococcus faecalis*** |  |  |
| **BM4110** | Recipient strain, Lin, Str | [80] |
|  |  |  |
| ***Streptococcus agalactiae*** |  |  |
| **UCN78** | Donor strain carrying Tn*916,* Tet | This study |
|  |  |  |
| ***Escherichia coli*** |  |  |
| **M15[pREP4]** | Protein expression host carrying pREP4 plasmid, Kan | Qiagen |
| **M15[pREP4]/pQE30Ω*asrR*** | M15[pREP4] carrying pQE30Ω*asrR for* AsrR expression, Amp, Kan | This study |
| **Top10** | Cloning strain | Invitrogen |
| **Top10/pCR2.1Ω*ohr-prom*** | Top10 carrying pCR2.1Ω*ohr-prom* for *ohr* D4-labelling | This study |
| **Amp, ampicillin; Ery, erythromycin; Fus, fusidic acid; Kan, kanamycin; Lin, lincomycin; Rif, rifampin; Str, streptomycin; Tet, tetracycline.** | | |
