# Supplementary material for: AsrR Is an Oxidative Stress Sensing Regulator Modulating Enterococcus faecium Opportunistic Traits, Antimicrobial Resistance, and Pathogenicity
Source: PLoS Pathog. 2012 Aug 2;8(8):e1002834. doi: 10.1371/journal.ppat.1002834 (PMC3410868; doi:10.1371/journal.ppat.1002834)
Supplement: Table S2 — Oligonucleotide primers used in this study. (DOC) [file ppat.1002834.s007.doc]

**Table S2.** **Oligonucleotide primers used in this study.**

| **Primer**^α^ | **Sequence** | **Position (5'-3')**^β^ or role | **Source** |
| --- | --- | --- | --- |
| Mutagenesis |  |  |  |
| *asrR*-DC1-F | CGGCCATGGACAATTGTTTGCAGCTGGTTTC | 365-396 | This study |
| *asrR*-DC2-R | CGCGGATCCTAGCTCTTTCACTGAAGTC | 887-859 | This study |
| *asrR*-DC3-F | CGCGGATCCCTTGAACAGGAAGAATTG | 1058-1085 | This study |
| *asrR*-DC4-R | CGGCCATGGAAGCATCGGGATATACTTTGTG | 1571-1540 | This study |
| *asrR*-control-R | CAATAACGGACTGGATCATA | 1499-1579 | This study |
| *asrR*-pOri23-F | CCGGTCGACAGAGGCAGCGCATAAAGTGT | 560-589 | This study |
| *asrR*-pOri23-R | CCGCTGCAGCGGTCAAATCGACAACCTCC | 1317-1288 | This study |
|  |  |  |  |
| RACE-PCR |  |  |  |
| sp1-*asrR*-R | GGTCAGATGTTGCAGTGT | 1106-1088 | This study |
| sp2-*asrR-*R | TTGCAACGCAGCACCTTGA | 1022-1003 | This study |
| sp3-*asrR*-R | CATCACCCGTTTCACGCTGAC | 975-954 | This study |
| sp1-*ohr*-R | TGTGCACTGACAGTAGAAGC | 473-453 | This study |
| sp2-*ohr*-R | GCACTGTTAAAACATGCACTGA | 416-394 | This study |
| sp3-*ohr*-R | CGTTTACCAGGAGCTGCAAT | 350-330 | This study |
|  |  |  |  |
| Protein expression |  |  |  |
| AsrR-F | GCGGATCCATGAGAGAAGATCTATATTTAGTTAATCAAG | 699-738 | This study |
| AsrR-R | GGGGTACCTCAGCCCTTTTTCTGGTCTTG | 1159-1130 | This study |
|  |  |  |  |
| Promoter region (EMSA) |  |  |  |
| Prom-*ohr*-F | CATCAGGTCCATCGTGAAGT | 1-20 | This study |
| Prom-*ohr*-R | TCCGCCTGTGTTGATCATTT | 285-265 | This study |
| Prom-*asrR*-F | TAGAGGCAGCGCATAAAGTG | 568-588 | This study |
| Prom-*asrR*-R | TAGGTAAGATTCAGCGGCTTG | 817-797 | This study |
|  |  |  |  |
| qRT-PCR |  |  |  |
| *asrR*-F | AACGGGTGATGAAAGAATCG | 965-984 | This study |
| *asrR*-R | GCATTTTTCGGGTCAGATGT | 1116-1097 | This study |
| *ohr*-F | AGGCGGACGCTCTGGAGAA | 279-297 | This study |
| *ohr*-R | TGCACTGAAACCAGCTGCAAA | 402-382 | This study |
| *ohr*-F2 | GCGGACGCTCTGGAGAAGT | 281-299 | This study |
| *ohr*-R2 | GCATTTTCGACGCGTTTACC | 361-342 | This study |
| *tuf*-F | TACACGCCACTACGCTCAC | NA | This study |
| *tuf*-R | AGCTCCGTCCATTTGAGCAG | NA | This study |
| *kat*-F | GGCCAATATGGCGAGATGAG | NA | This study |
| *kat*-R | TGGCCGATTTCTTCTGTTGC | NA | This study |
| *sigV*-F | ATTGATCCGCACGGCATTAG | NA | This study |
| *sigV*-R | ATGGAGCTCTGCAACGGAAG | NA | This study |
| *uvrA*-F | CCCACCAGAACCGACAAAAG | NA | This study |
| *uvrA*-R | TGGCTACTTGTTCCGCCATT | NA | This study |
| *gpx*-F | GCTCCTGGTACTGTCACTAAAGCA | NA | This study |
| *gxp*-R | TTTGTGGTGCAAATCGTTCG | NA | This study |
| *acm*-F | AGAACCAAGACGGCAAACGA | NA | This study |
| *acm*-R | TGAAACGATAGCGCCAATCA | NA | This study |
| *pbp5*-F | TCGGCGAAACAGCAGTACAA | NA | This study |
| *pbp5-*R | GCCAACGGAATCCCTAAAGC | NA | This study |
|  |  |  |  |
| Cotranscription of *ohr* and *asrR* |  |  |  |
| Cotrs-*ohr*-F | AGGCGGACGCTCTGGAGAA | 278-296 | This study |
| Cotrs-*ohr*-R | ACACTTTATGCGCTGCCTCT | 588-569 | This study |
| Cotrs-*asrR*-F | ACTACACTTCCCTCCTCAAGCCG | 780-802 | This study |
| Cotrs-*asrR*-R | CGGGTCAGATGTTGCAGTGTATCC | 1107-1084 | This study |
| Intergenic-F | AGAGGCAGCGCATAAAGTGTG | 569-589 | This study |
| Intergenic-R | ACTGGGTATAGGTAAGATTCAGC | 824-802 | This study |
| Control-F | CATCAGGTCCATCGTGAAGTAAG | NA | This study |
| Control-R | ACACTTTATGCGCTGCCTCT | 588-569 | This study |
|  |  |  |  |
| Various |  |  |  |
| pG(+)host9-F | GTTGGGTAACGCCAGGGTTT | Cloning verification | This study |
| pG(+)host9-R | CTACTGACAGCTTCCAAGGAG | Cloning verification | This study |
| M13-F | GTTGTAAAACGACGGCCAGT | Cloning verification | Invitrogen |
| M13-R | AGCGGATAACAATTTCACAC | Cloning verification | Invitrogen |
| pQE30-F | GGAGAAATTAACTATGAGAGG | Cloning verification | Qiagen |
| pQE30-R | GTTCTGAGGTCATTACTGG | Cloning verification | Qiagen |
| pCR2.1-D4 | [D4-PA]GTTGTAAAACGACGGCCAGT | D4 labelling of inserts | This study |
| TetM-F | AGTTTTAGCTCATGTTGATG | *tetM* screening | [81] |
| TetM-R | TCCGACTATTTAGACGACGG | *tetM* screening | [81] |
| ^α^ F for forward and R for reverse. ^β^ Position from the HM1070 *ohr* and *asr* cluster sequence (Genbank accession number JQ390466).  NA, Not Applicable | | | |
